# Hepatocellular carcinoma risk reduction associated with antiviral treatment in patients with noncirrhotic chronic hepatitis B: a systematic literature review and comparative meta-analysis

**DOI:** 10.1186/s12876-026-04996-y

**Published:** 2026-06-26

**Authors:** Arpan Mohanty, Alice E. Stead, Laura E. Telep, Amanda W. Singer, Tristan Curteis, Anna Zolotor, Catherine Frenette, Dona Khoshabafard, Kyle Hammond, Tom McQuaid, Robin K. Kelley

**Affiliations:** 1 Boston University Chobanian and Avedisian School of Medicine and Boston Medical Center, 85 East Concord Street, Boston, Massachusetts 02118 USA; 2 Gilead Sciences, Inc., 333 Lakeside Drive, Foster City, California 94404 USA; 3 Costello Medical, Ltd, 1 New York Street, Manchester, M14HD UK; 4 Costello Medical, Inc, 3 Center Plaza, Boston, Massachusetts 02108 USA; 5 Helen Diller Family Comprehensive Cancer Center, University of California, 550 16th Street, San Francisco, California 94158 USA

**Keywords:** Antiviral therapy, Chronic hepatitis B, Hepatocellular carcinoma, Meta-analysis, Systematic review

## Abstract

**Background:**

Chronic hepatitis B (CHB) affects nearly 300 million people worldwide and is a leading cause of hepatocellular carcinoma (HCC). While cirrhosis is a risk factor for HCC, hepatitis B virus (HBV) DNA integration can lead to HCC in non-cirrhotic individuals. This study synthesized existing data to compare HCC risk among non-cirrhotic patients treated with antivirals versus those untreated.

**Methods:**

A systematic literature review was conducted. Databases were searched in February 2023 and handsearches were performed to identify studies reporting HCC incidence in non-cirrhotic CHB patients, both treated with antivirals for a mean or median duration of at least three years and untreated. A meta-analysis compared HCC incidence rates between treated and untreated populations.

**Results:**

Two thousand, one hundred sixty-four records were screened and 61 publications on 50 studies were included. HCC cumulative incidence rates increased with greater follow-up, ranging from 0 to 3% at one year and 0 to 30% at 10 years. Incidence rates ranged from 0 per 100 to 2.43 per 100 person-years. The comparative meta-analysis of four studies indicated that treated populations had a 40% reduction in HCC incidence compared to untreated groups (incidence rate ratio [IRR]: 0.59; 95% CI: 0.50, 0.69), with incidence rates expressed per 100 person-years. Sensitivity analyses confirmed the robustness of these findings.

**Conclusions:**

Antiviral therapy significantly reduces HCC risk in non-cirrhotic CHB patients. These results, with other literature, support treatment guideline expansion to include non-cirrhotic individuals and prevent HCC development. Further research is necessary to evaluate the impact of early versus late treatment initiation and explore potential differences between antivirals.

**Systematic review registration:**

PROSPERO ID: CRD42023396260. Date of registration: 11 April 2023.

**Supplementary Information:**

The online version contains supplementary material available at https://doi.org/10.1186/s12876-026-04996-y.

## Introduction

Chronic hepatitis B (CHB), defined as the presence of hepatitis B surface antigen (HBsAg) for at least 6 months after hepatitis B virus (HBV) infection, is a significant global health challenge that affects nearly 300 million people worldwide [1]. CHB is assessed through key markers such as HBsAg, HBV DNA levels, alanine aminotransferase (ALT), and liver disease progression. Hepatitis B e-antigen (HBeAg), also used in CHB assessment, is a marker of HBV replication, while the presence of antibodies against HBeAg is an equally important marker of partial immune control [2]. Long-term complications of CHB infection include liver failure, advanced liver disease, and hepatocellular carcinoma (HCC), all of which are associated with high morbidity and mortality [3]. CHB accounts for over half of HCC cases globally [4]. Cirrhosis is a well-established risk factor for HCC across all major causes of liver diseases, including chronic viral hepatitis [5, 6]. However, HBV is unique in that early host cell chromosomal integration of viral DNA contributes to HCC development even in non-cirrhotic individuals, with HBV DNA integrations found in approximately 80% of HCC tumor tissue [7]. 

HBV is designated as a group 1 carcinogen by the International Agency for Research on Cancer (IARC) [8] and is carcinogenic and mutagenic to host cell DNA [7]. HBV can drive oncogenesis through the production of viral proteins that disrupt normal cell growth, through DNA integrations which facilitate chromosomal translocations, and by promoting inflammation that results in cell turnover and clonal expansion [7]. These processes contribute to genomic instability and malignant transformation [7, 9]. There is evidence that viral DNA integration and concomitant chromosomal translocations occur within hours of infection, and initial studies evaluating the impact of antiviral treatment have shown reduction of integrations and reduced expansion of mutant clone populations [10, 11]. This likely explains in part why long-term antiviral therapy with nucleos(t)ide analogues (NAs) has demonstrated the ability to mitigate HCC risk, particularly when initiated early in the disease course and maintained over a significant duration of time [12, 13]. The critical role of DNA damage in malignant transformation and tumor progression underscores the need for early interventions to counteract HBV’s mutagenic potential and mitigate HCC risk.

Current international treatment guidelines from the European Association for the Study of the Liver (EASL) [2], the American Association for the Study of Liver Diseases (AASLD) [14], and the Asian Pacific Association for the Study of the Liver (APASL) [15] recommend initiating antiviral therapy in cirrhotic individuals with CHB. The EASL 2025 guidelines recognize the potential benefits of earlier treatment in all HBV DNA-positive individuals to prevent viral replication and subsequent liver damage, while acknowledging there is less conclusive evidence supporting a significant reduction in HCC risk with NA therapy in non-cirrhotic populations due to limited long-term follow-up data and low event incidence [2]. Overall, treatment guidelines continue to focus primarily on treating cirrhotic individuals with CHB, while guidelines for management of non-cirrhotic individuals are more complex and take into consideration factors such as viral load and ALT values [7]. High-quality synthesis of evidence of HCC risk reduction attributable to antiviral therapy may elucidate the benefits of treatment expansion for individuals with CHB, specifically in non-cirrhotic, immune-tolerant, or younger individuals earlier in disease.

Conducting randomized controlled trials (RCTs) to evaluate the efficacy of antiviral therapy in reducing HCC incidence is potentially unethical and impractical due to the long latency period for HCC development, which requires extended follow-up, incurs high costs, and faces challenges due to heterogeneous and evolving clinical practices. Thus, this *de novo*, clinically focused systematic literature review (SLR) considers both RCTs and observational studies to identify the incidence rates of HCC in individuals with non-cirrhotic CHB, stratified by those treated and untreated with antivirals. A meta-analysis was performed to directly compare the incidence of HCC between individuals who were treated with antiviral therapies and individuals who were untreated within the same study to evaluate the hypothesis that antiviral treatment reduces the long-term risk of HCC in non-cirrhotic populations. This study is intended to help inform clinical decision-making for individuals with non-cirrhotic CHB by examining the benefits of initiating antiviral treatment prior to progression to advanced liver disease.

## Methods

### Search strategy and selection criteria

The SLR was conducted according to a prespecified protocol which was registered in PROSPERO (ID: CRD42023396260). Searches were performed in February 2023 across electronic databases including MEDLINE, MEDLINE In-Process, MEDLINE Epub Ahead of Print, Embase, The Cochrane Database of Systematic Reviews (CDSR) and the Cochrane Central Register of Controlled Trials (CENTRAL), restricting results to literature published from 2002 onwards, to identify primary research relating to the study objective. The search strategies used are shown in Tables S1-S4. Following the initial searches, Ovid, which was used to search MEDLINE, MEDLINE In-Process, MEDLINE Epub Ahead of Print, and Embase, announced that some Embase records had been missing. An additional search was performed in March 2023 to identify relevant records which were missing from the Ovid platform during the initial searches (Table S5).

The bibliographies of all relevant SLRs and network meta-analyses (NMas) included from the database searches were hand-searched, as were select conference proceedings from the previous 2 years, to identify any additional relevant studies (Table S6).

Each title and abstract, and subsequently each full-text article, was assessed for inclusion by two independent reviewers. Discrepancies in inclusion were resolved through discussion until consensus was reached, with a third independent reviewer making the final decision if necessary. Studies eligible for inclusion in the SLR were peer-reviewed, published in English, reported HCC incidence in children or adults with non-cirrhotic CHB based on any combination of clinical assessment, non-invasive testing/imaging, or biopsy, and included populations untreated with antivirals or treated with antivirals for at least 3 years, reported as either mean or median treatment duration (excluding mixed cohorts), with any of the following interventions: tenofovir alafenamide (TAF)/Vemlidy^®^, tenofovir disoproxil fumarate (TDF)/Viread^®^, entecavir (ETV)/Baraclude^®^, telbivudine (LdT)/Tyzeka^®^, adefovir dipivoxil/Hepsera^®^, clevudine/Levovir^®^, or lamivudine (LAM)/Epivir-HBV/Zeffix/Heptodin^®^. Included records were examined for overlapping populations prior to data extraction.

### Source data analysis

Data extraction for included studies was performed in line with guidelines from the York University Centre for Reviews and Dissemination (CRD) [16]. Study characteristics, baseline characteristics of the patient population, and relevant efficacy, safety, and patient-reported outcomes were extracted into a pre-specified Microsoft Excel extraction grid by one reviewer, and verified by a second, independent reviewer. Any discrepancies were discussed and, if necessary, a third individual was enlisted to arbitrate the final decision. The quality of included studies was assessed by one individual and verified by a second using the assessment tool developed by the Joanna Briggs Institute (JBI) for prevalence data, modified to account for the diagnosis of HBV infection and HCC in the patient population (Table S8) [17]. 

Based on studies identified in the SLR, the feasibility of performing meta-analyses to evaluate the impact of longer-term antiviral treatment on the risk of HCC in individuals with non-cirrhotic CHB, as well as simply the reduction in HCC risk among treated versus untreated populations, was assessed. A thorough assessment of heterogeneity investigated differences in study, patient, and treatment characteristics among the included studies. Additionally, comprehensive investigations of available data on the incidence of HCC were carried out, with both cumulative incidence and incidence rates explored.

Based on the results of the feasibility assessment, an unadjusted meta-analysis was performed on a subset of studies reporting on both treated and untreated populations of patients with CHB to estimate the incidence rate ratio (IRR) and incidence rate difference (IRD) of HCC between patients treated with NAs (who were treatment-naïve at baseline) and untreated patients. Reported (and, where appropriate, derived) study-level values for person-time and number of HCC events were used as input data for the meta-analysis. Methods for derivation of meta-analysis inputs are shown in Supplementary File 1.

The *meta* package (version 6.5-0) was used in R (version 4.3.0) [18, 19]. The *metainc* function was used for this analysis with both fixed effect and random effects estimates produced for the IRR and the IRD. The default settings of the *meta* package were used. The Mantel-Haenszel method was used for pooling [20], and 95% confidence intervals (CIs) were generated for each estimate. I^2^ was calculated to assess statistical heterogeneity across studies. In order to identify potential outliers, a Baujat plot was generated using the *baujat* function in the *meta* package [21]. A sensitivity analysis was then conducted to investigate the results of the direct comparison meta-analysis excluding an identified outlier.

## Results

### SLR study characteristics

A total of 2,803 records were retrieved by the electronic database searches. After de-duplication of results, 2,164 were suitable for review. After title and abstract review, 319 records were selected for full text review. Of these, 56 fulfilled the eligibility criteria for inclusion. Supplementary searches of congresses and SLR bibliographies identified five additional records. In total, 61 publications reporting on 50 unique studies were included in the SLR, shown in the Preferred Reporting Items for Systematic Reviews and Meta-Analyses (PRISMA) diagram (Fig. S1).

A summary of study characteristics of included studies is shown in Table 1. The majority were retrospective cohort studies (*n* = 33), while 11 were prospective cohort studies, three were national database/registry studies, three were RCT open-label extension studies, and one was an RCT. Tseng 2017 included both retrospective and prospective cohort designs [64]. Most studies (*n* = 39) were conducted in Asia, with South Korea being the most represented country (*n* = 16 studies). Japan (*n* = 9) and Taiwan (*n* = 8) were the second and third most frequently represented countries. The remaining studies were multinational (*n* = 7) or conducted in China (*n* = 6), Italy (*n* = 3), or France (*n* = 1). Sample sizes for included studies ranged from 39 to 25,940 participants, with 14 studies including 1,000 or more participants and only six studies reporting fewer than 100 participants. Most studies demonstrated appropriate methodology, with an adequate sample size in 49/50 studies and 44/50 studies using valid methods to identify HCC development (Table S8).

**Table 1:** Summary of study characteristics

Study	Study design	Country	Study period	Source of data	Treatments evaluated	Sample size^a^	Definition of cirrhosis status
Ando 2018 [22]	Retrospective cohort	Japan	Jan 2000–Jan 2013^b^	Nagoya University Hospital	LAM; LAM + ADV; ETV; ETV + ADV; TDF	*N* = 105	Cirrhosis was diagnosed by histological analysis and/or appearance of cirrhotic liver on ultrasonography, for example, superficial nodularity, coarse parenchymal echo pattern, signs of portal hypertension (splenomegaly > 120 mm, diameter of portal vein > 12 mm, patent collateral veins, or ascites), and/or the presence of gastroesophageal varices on endoscopy
Chiu 2020 [23]	Retrospective cohort	Taiwan	Jan 2007–Jan 2016^b^	Chang Gung Memorial Hospital	No treatment (ETV or TDF cessation)	*N* = 460	Noncirrhosis was diagnosed based on biopsy, Fibrosis-4 or liver stiffness measurement at baseline, combined repeated ultrasound findings without clinical features, such as splenomegaly, ascites, or gastroesophageal varices at baseline
Cho 2014 [24]	Retrospective cohort	South Korea	Jan 2007–Jan 2012^b^	Samsung Medical Center	ETV; LAM; CLV; sequential or combination NA therapy; no treatment	*N* = 1,817	Cirrhosis was determined by liver biopsy or imaging modalities with findings of irregular surface nodularity, parenchymal or morphological abnormality and manifestations of portal hypertension designated as liver cirrhosis
Choi 2020 [25]	Retrospective cohort	South Korea	Jan 2007–Dec 2016^c^	Asan Medical Center	ETV; TDF	*N* = 2,612	Cirrhosis was defined by the presence of any of the following findings: coarse liver echotexture and nodular liver surface on ultrasonography, clinical features of portal hypertension (e.g., ascites, splenomegaly, or varices), or thrombocytopenia (< 150,000/mm^3^)
Choi 2022a [26]	Retrospective cohort	South Korea	Jan 2000–Apr 2020^c^	Asan Medical Center; Seoul National University Hospital; Severance Hospital; Samsung Medical Center	ETV; TDF; no treatment	*N* = 2,073	Cirrhosis was defined as the presence of any of the following characteristics: coarse liver echotexture and nodular liver surface on ultrasonography, clinical features of portal hypertension (e.g., ascites, splenomegaly, and varices), and platelet count below 100,000/µL
Choi 2022b [27]	Retrospective cohort	South Korea; Hong Kong; Taiwan	NR	NR	ETV; TDF	*N* = 7,545	NR
Chun 2021 [28]	National registry/ database	South Korea	Jan 2012–Dec 2018^c^	NR	ETV; TDF	*N* = 7,602	Cirrhosis was defined as ICD-10 code K74
Dezanet 2021 [29]	Prospective cohort	France	Jan 2002–Jan 2018^c^	Four centers in France	TDF-based ART; TAF-based ART^e^	*N* = 155	Liver fibrosis was assessed using the FibroTest^®^ calculated from standard battery of biochemical markers. Scores ranged from 0 to 1. METAVIR equivalents of this measure, as established in the HIV-HBV coinfected population, were used to grade liver fibrosis (F2: 0.48–0.58, F3: 0.59–0.73, F4: ≥0.74)
Hosaka 2010 [30]	Retrospective cohort	Japan	Sep 1995–Sep 2007^b^	Toranomon Hospital	LAM + ADV	*N* = 186	Cirrhosis was diagnosed by laparoscopy or liver biopsy, and in the patients who had not received them, by clinical data, imaging modalities, and portal hypertension
Hosaka 2013 [31]	Retrospective cohort	Japan	Jan 1973–Dec 2011^c^	Toranomon Hospital	ETV; LAM; no treatment	*N* = 601	Cirrhosis was determined based on laparoscopy, liver biopsy, imaging modalities, or portal hypertension
Hosaka 2022 [32]	Retrospective cohort	Japan	NR–Dec 2020^d^	Toranomon Hospital	ETV	*N* = 132	Cirrhosis was determined by laparoscopy, liver biopsy, imaging modalities, or portal hypertension
Ji 2022 [33]	RCT open-label extension	China	Nov 2013–NR	9 first-class hospitals (not specified)	ETV; ETV+Biejia-Ruangan	*N* = 500	NR
Kim 2015 [34]	RCT open-label extension	Multinational	Jun 2005–Jan 2016	NR	TDF; ADV followed by TDF	*N* = 482	Cirrhosis was defined as Ishak fibrosis scores of 5 or 6
Kim 2017a [35]	Retrospective cohort	South Korea	Jan 2007–Dec 2015^c^	Asan Medical Center	ETV	*N* = 850	Cirrhosis was diagnosed based on histological and/or radiographical findings with evidence of portal hypertension (e.g., splenomegaly, ascites, or varices) in the presence of significant thrombocytopenia (platelet count < 150,000/mm^3^)
Kim 2017b [36]	Retrospective cohort	South Korea	Jan 2007–Dec 2015^c^	Samsung Medical Center	ETV	*N* = 432	Cirrhosis was clinically defined when patients showed cirrhotic configuration of the liver on imaging studies (nodular liver surface or caudate lobe hypertrophy) with thrombocytopenia (< 150 × 10^3^/L) or splenomegaly (by imaging) and/or by the presence of varices (by upper endoscopy or imaging studies)
Kim 2018 [37]	Retrospective cohort	South Korea	Jan 2007–Apr 2017^d^	Ulsan University Hospital	ETV; TDF	*N* = 198	Cirrhosis was determined via liver biopsy or clinically defined in the absence of liver biopsy based on repeated liver imagingstudies (nodular liver surface or caudate lobe hypertrophy) with thrombocytopenia (< 150 × 1000/mm^3^) or splenomegaly (by imaging), and/or by the presence of varices (by upper endoscopy or imaging studies)
Ko 2019 [38]	Prospective cohort	Hong Kong	Jan 2002–Dec 2015^d^	Queen Mary Hospital	ETV	*N* = 1,033	Cirrhosis was defined by the following combined parameters: (a) ultrasonographic evidence of small-sized liver with or without development of splenomegaly/ascites/varices; and (b) APRI = (AST/upper limit of normal)/platelet count (x10^9^L) x 100) greater than 2
Kobashi 2011 [39]	Prospective cohort	Japan	Nov 2000–Nov 2010^d^	NR	ETV; LAM	*N* = 194	Cirrhosis diagnosis was confirmed by liver histology, clinical signs (encephalopathy or ascites), evidence of esophagogastric varices by endoscopy, or imaging modalities such as ultrasonography, CT, or MRI
Kozuka 2019 [40]	Retrospective cohort	Japan	Sep 2006–June 2017^c^	Osaka City University Hospital	ETV	*N* = 105	Liver cirrhosis was diagnosed by histological examination according to the METAVIR scoring system using imaging modalities such as ultrasonography, CT, or MRI and by signs of portal hypertension
Lampertico 2007 [41]	RCT open-label extension	Italy	Jan 2001–NR	Hospital (not specified)	LAM + ADV	*N* = 39	Cirrhosis was defined on clinical grounds, that is, albumin level < 3.5 g/dL, platelet count < 100,000 mm3, clinical decompensation, and/or ultrasound demonstration of surface nodularity, splenomegaly, and > 15-mm portal vein diameter
Lee 2016 [42]	Retrospective cohort	South Korea	Jan 2009–Dec 2012^b^	Bundang Jesaeng Hospital	ETV	*N* = 66	Cirrhosis was diagnosed based on ultrasonographic findings
Lee 2019 [43]	RCT	South Korea	Dec 2008–Apr 2015	18 medical centers (not specified)	ETV; LAM; ETV + ADV;^e^ ETV+tenofovir;^e^ LAM + ADV;^e^ LAM+tenofovir^e^	*N* = 72	Advanced fibrosis and cirrhosis were confirmed according to grades F3 and F4, or clinical evidence of overt cirrhosis (gastroesophageal varices identified on endoscopy, or platelet count < 100,000/mm3 and splenomegaly [> 12 cm]) with a high viral load (HBV DNA 2,000 IU/mL)
Lee 2021 [44]	National registry/ database	South Korea	Jan 2012–Dec 2014^d^	Hospital (not specified)	TDF; TDF-based combination therapy	*N* = 2,030	Cirrhosis was defined as patients with ICD-10 K74 code
Lee 2022 [45]	Retrospective cohort	South Korea	Jan 2007–Dec 2020^b^	Korea University Guro Hospital; Korea University Ansan Hospital; Gacheon University Gil Medical Center; Wonju Severance Christian Hospital	ETV; TDF	*N* = 787	Cirrhosis was diagnosed based on ultrasonographic findings including undulation of liver surfaces, blunt liver edge, severe coarse liver echotexture, liver shrinkage, splenomegaly, or ascites
Li 2017 [46]	Prospective cohort	China	Mar 2012–May 2015	First Affiliated Hospital of Zhengzhou University	ETV; LAM; ADV; LAM + ADV	*N* = 965	NR
Lim 2014 [47]	Retrospective cohort	South Korea	Nov 1999–Mar 2013^c^	Asan Medical Center	LAM; ETV	*N* = 878	Cirrhosis was diagnosed when the liver echotexture was coarse and nodular and/or when features of portal hypertension (e.g., ascites, splenomegaly, and varices) were noted
Lin 2019 [48]	Retrospective cohort	Taiwan	Jan 2009–Dec 2010^b^	Mackay Memorial Hospital	ETV; LAM; LdT	*N* = 270	Patients underwent abdominal ultrasonography to determine the changes of cirrhosis
Moon 2015 [49]	Retrospective cohort	South Korea	Jan 2003–Dec 2012^d^	Chonbuk National University Hospital	LAM; LdT; CLV; ADV;^e^ ETV;^e^ ETV + ADV;^e^ TDF^e^	*N* = 330	Cirrhosis diagnosis was based on liver biopsies or radiologic findings with or without varices at upper gastrointestinal endoscopy. Radiologic findings of cirrhosis were defined as coarse liver echotexture with parenchymal nodularity, irregular surface nodularity, and the features of portal hypertension
Nagata 2016 [50]	Retrospective cohort	Japan	Dec 1999–Jun 2010^b^	Tokai University Hospital	LAM; ETV; no treatment (LAM or ETV cessation)	*N* = 94	NR
Niro 2013 [51]	Prospective cohort	Italy	1998–2007^b^	Casa Sollievo Sofferenza Hospital	LAM; ETV; ADV; LdT; tenofovir; LAM + ADV	*N* = 74	To define grading and staging of liver disease, a liver biopsy was advised before commencing antiviral treatment. Histological activity was evaluated by the modified Scheuer’ score, which grades the inflammatory activity on a scale from 0 to 3 and fibrosis from 0 to 4. In patients who did not consent to liver biopsy, cirrhosis was diagnosed with indirect criteria, such as platelet count ≥ 100,000 mm^3^, ultrasound features of surface nodularity, splenomegaly and portal vein diameter > 13 mm, and/or the endoscopic detection of esophageal varices
Oh 2020 [52]	Retrospective cohort	South Korea	Jan 2011–Dec 2015^b^	9 academic hospitals (not specified)	ETV; TDF	*N* = 935	The severity of fibrosis was calculated using the fibrosis-4 score (e.g., no fibrosis if the fibrosis-4 scores were < 1.45; possible fibrosis if the fibrosis-4 scores were between 1.45–3.25; and advanced fibrosis if the fibrosis-4 scores were > 3.25). Cirrhosis was defined based on the radiologic features of cirrhosis (e.g., undulating liver surface, compensatory left lobe hypertrophy, splenomegaly, and portosystemic shunts, and so forth)
Orito 2015 [53]	Retrospective cohort	Japan	NR	15 hospitals in the Japanese Red Cross Liver Network	ETV; LAM; LAM+ADV^e^	*N* = 492	NR
PAGE-B Cohort [54]	Prospective cohort	Greece; Spain; Germany; Italy; The Netherlands; Turkey	NR–Dec 2012^b^	10 treatment centers (not specified)	ETV; TDF; ETV + ADV; ETV + TDF; TDF + LAM; TDF + LdT	*N* = 1,023	Elastographic cirrhosis was considered to be present in cases with reliable liver stiffness measurements of ≥ 12 kPa, which represents the lowest LSM cut-off for the diagnosis of cirrhosis according to the international guidelines
Papatheodoridis 2015 [55]	Retrospective cohort	Greece; Spain; Italy; The Netherlands; Turkey	NR–Oct 2013^d^	7 liver clinics (not specified)	ETV; TDF; ETV + ADV; ETV + TDF; TDF + LAM; TDF + LdT	*N* = 1,085	Patients were classified according to the severity of their liver disease into (a) patients with CHB only if they had a pretreatment liver biopsy without lesions of cirrhosis, (b) patients with compensated cirrhosis if they had histological findings and/or ultrasonographic findings (nodules in the hepatic parenchyma, spleen > 12 cm, portal vein > 16 mm) and/or endoscopic findings of cirrhosis (varices, portal gastropathy) and (c) patients with decompensated cirrhosis if they had developed ascites, variceal bleeding, encephalopathy and/or non-obstructive jaundice (bilirubin >3 mg/dl). Patients without a pretreatment liver biopsy and without any other sign of cirrhosis were considered as cases with unclassified disease severity
Papatheodoridis 2017 [56]	Prospective cohort	Greece; Spain; Germany; Italy; The Netherlands; Turkey	NR–Apr 2016^d^	10 liver clinics (not specified)	ETV; TDF; ETV + ADV; ETV + TDF; TDF + LAM; TDF + LdT	*N* = 1,379	The severity of liver disease was classified into CHB only (without cirrhosis) according to findings from liver biopsies and CHB with compensated cirrhosis according to histological, elastographic (reliable liver stiffness measurement > 14 kPa), ultrasonographic, and/or endoscopic findings. Patients without a pretreatment liver biopsy and without any other sign of cirrhosis were considered as cases with unclassified disease severity
Papatheodoridi 2022 [57]	Retrospective cohort	Greece; Taiwan	Jan 2011–Jan 2017^b^	NR	Patients who discontinued NA therapy (not specified); ETV; LAM; TDF	*N* = 490	Absence of cirrhosis was defined as Ishak staging score of ≤ 4 or liver stiffness ≤ 10 kPa at transient elastography [58]
Pellicelli 2014 [59]	Retrospective cohort	Italy	Jan 1998–Dec 2012^c^	Liver units or gastroenterology treatment centers that were part of the CLEO group	LAM; ETV; ADV; LdT; TDF; LAM + ADV	*N* = 193	Cirrhosis diagnosis was based on histological criteria (Ishak stages 5 and 6). In patients that did not undergo liver biopsy, cirrhosis was diagnosed on the basis of other criteria, such as ultrasound signs (spleen size > 12 cm, coarse nodular eco-pattern in the hepatic parenchyma), and/or transient elastometry value (FibroScan^®^) > 12.5 kPa and/or endoscopic findings compatible with cirrhosis (esophageal varices, portal gastropathy), and/or platelet count < 100,000 mm^3^
RETRACT-B [60]	Prospective cohort	Belgium; Germany; Greece; The Netherlands; Spain; Hong Kong; Taiwan; Canada	Jan 2001–Jan 2020^b^	13 treatment centers (not specified)	Patients who discontinued NA therapy (not specified)	*N* = 1,368	Cirrhosis was diagnosed based on histologic findings or ultrasonographic evidence with or without splenomegaly
Shim 2017 [61]	Retrospective cohort	South Korea	Jan 2007–June 2016^c^	Kyung Hee University Hospital	ETV	*N* = 133	Cirrhosis was diagnosed with imaging, endoscopic, or histologic examinations. Typical imaging findings were an irregular nodular surface, coarse parenchymal echogenicity reflecting underlying nodularity, and/or caudate lobe hypertrophy. The presence of esophageal or gastric varices and ascites was also included as a diagnostic criterion for cirrhosis
Shin 2018 [62]	Retrospective cohort	South Korea	Jan 2007–May 2017^d^	Ulsan University Hospital in Korea	ETV	*N* = 454	Cirrhosis was determined via liver biopsy, or clinically defined in the absence of liver biopsy based on repeated liver imaging studies (nodular liver surface or caudate lobe hypertrophy) with thrombocytopenia (< 150 K/µL) or splenomegaly (by imaging), and/or by the presence of varices (by upper endoscopy or imaging studies)
Sou 2020 [63]	Retrospective cohort	Taiwan	Jan 2007–Oct 2012^b^	Kaohsiung Chang Gung Memorial Hospital or China Medical University Hospital	ETV	*N* = 890	Cirrhosis was diagnosed according to either (1) histology or (2) repeated ultrasounds with consistent findings suggestive of cirrhosis in addition to clinical features such as splenomegaly, thrombocytopenia, ascites, or gastroesophageal varices
Tseng 2017 [64]	Retrospective cohort and prospective cohort	Taiwan	Jan 1985–Dec 2015^c^	ERADICATE-B study; National Taiwan University Hospital; Taipei Tzuchi Hospital	NA treatment (not specified); no treatment	*N* = 2,607	Cirrhosis at baseline was diagnosed either by histology, APRI value > 2.0, or ultrasonographic findings together with clinical features such as thrombocytopenia, gastroesophageal varices, or ascites
Wang 2016 [65]	Retrospective cohort	Taiwan	Oct 2011–May 2013^d^	Chang Gung Memorial Hospital	TDF	*N* = 144	Cirrhosis was diagnosed on the basis of liver stiffness measurement and/or if repeated ultrasound and clinical findings were suggestive of cirrhotic conditions, such as splenomegaly, thrombocytopenia, varices, ascites, and hepatic encephalopathy
Wong 2013 [66]	Prospective cohort	Hong Kong	Dec 2005–Aug 2012^b^	Hepatitis clinics at Prince of Wales Hospital	ETV	*N* = 1,199	Cirrhosis was defined as a shrunken small liver with a nodular surface noted on imaging oft he liver and clinical features of portal hypertension (e.g., ascites, splenomegaly, and varices)
Wu 2017 [67]	Retrospective cohort	Taiwan	Jan 2007–Jan 2014^d^	Chang Gung Memorial Hospital	TDF; ETV	*N* = 296	Cirrhosis was diagnosed according to repeated ultrasounds suggestive of cirrhosis in addition to clinical features such as splenomegaly, thrombocytopenia, ascites or varices
Yamada 2015 [68]	Retrospective cohort	Japan	Jul 2004–Jul 2012^b^	Osaka University Hospital; Osaka Liver Forum	ETV	*N* = 404	Cirrhosis was defined by a shrunken, small liver with nodular surface as noted on liver imaging and by clinical features of portal hypertension
Yang 2013 [69]	Retrospective cohort	Taiwan	Aug 2006–May 2010^d^	Chang Gung Memorial Hospital	ETV	*N* = 314	Cirrhosis was diagnosed by: (i) liver biopsy histology or (ii) combined repeated ultrasound findings suggestive of cirrhosis from at least two examinations performed a minimum of 6 months apart with clinical features such as splenomegaly, thrombocytopenia, esophageal varices, ascites or hepatic encephalopathy
Yang 2014 [70]	Retrospective cohort	Taiwan	Jan 1990–Jan 2009^b^	National Taiwan University Hospital	LAM	*N* = 54	NR
Yip 2020 [71]	National registry/database	Hong Kong	Jan 2008–Dec 2018^c^	CDARS	ETV; TDF	*N* = 25,940	Cirrhosis was identified by ICD-9-CM diagnosis codes for cirrhosis and its related complications
Yuen 2007 [72]	Prospective cohort	Hong Kong	Jun 1994–Aug 1997^b^	Queen Mary Hospital	LAM; famciclovir followed by LAM; no treatment	*N* = 266	Cirrhosis development was defined as ultrasonographic evidence of small-sized liver with or without splenomegaly and ascites

*Abbreviations*: *ADV* adefovir, *ALT* alanine transaminase, *APRI* AST to platelet ratio, *ART* antiretroviral therapy, *AST* aspartate aminotransferase, *CLEO* Club Epatologi Ospedalieri, *CLV* clevudine, *CT* computed tomography, *ETV* entecavir, *LAM* lamivudine, *LdT* telbivudine, *MRI* magnetic resonance imaging, *NA* nucleo(s/t)ide analog, *NR* not reported, *RCT* randomized control trial, *TAF* tenofovir alafenamide, *TDF* tenofovir disoproxil fumarate

^a^The sample size represents the number of patients included in each study who were relevant to this SLR, which may be a sub-population of the overall number of patients included in a given study

^b^These dates represent that study’s start and end of patient enrollment, as study start and end date were not reported

^c^These dates represented the start of patient enrollment, and the end of data collection, as the study start date and end date were not reported

^d^These dates represent the data collection start and end date, as study start date and end date were not reported

^e^Treatments were received during follow-up but not at baseline

### SLR baseline characteristics

The baseline characteristics extracted across studies included age, sex, race or ethnicity, prior and current treatments, ALT levels, HBeAg status, and coinfections (Table 2). Apart from Ji 2022 [33], all studies reported age at baseline, with mean age across reporting study cohorts ranging from 38.4 to 59.0 years. Sex was reported by 49 studies, revealing a predominantly male demographic ranging from 45.1% to 95.5% of study cohorts. Race or ethnicity was reported by 21 studies; five studies focused exclusively on white populations and eleven on Asian populations.

**Table 2:** Summary of baseline characteristics

Study	Cohort	Age	Sex	Race or ethnicity	Prior treatments	Current treatments	ALT	HBV DNA	HBeAg status	Coinfections
Ando 2018 [22]	Full study cohort (*N* = 133)	Median: 51 years (range 20–79)	Male: 79/133 Female: 54/133	NR	IFN: 0/133	NA: 133/133 (100%)	Median: 23 IU/L (range 8-107)	NR	Positive: 41/133 Negative: 92/133	No coinfections
Chiu 2020 [23]	ETV group (*n* = 303)	Mean: 51.3 years (SD 10.5)	Male: 244/303 Female: 59/303	Asian: 100%	ETV: 100%	No current treatments	Mean: 322.5 U/L (SD 487.6)	Mean: 5.52 log IU/ml (SD 1.52)	Negative: 100%	No coinfections
	TDF group (*n* = 157)	Mean: 52.1 years (SD 10.4)	Male: 126/157 Female: 31/157	Asian: 100%	TDF: 100%	No current treatments	Mean: 316.4 U/L (SD 443.9)	Mean: 6.03 log IU/mL (SD 1.60)	Negative: 100%	No coinfections
Cho 2014 [24]	NA-treated (*n* = 1,378)	Mean: 47.5 years (SD 10.5)	Male: 908 (65.9%)	NR	No prior treatments	CLV: 49 (3.5%); ETV: 1,002 (73.1%); LAM: 69 (5.0%); combination or sequential: 258 (18.7)%	Mean: 155 U/L (SD 276.8)	Mean: 6.38 log IU/mL (SD 1.50)	Positive: 54.9%	No coinfections
	Untreated inactive CHB (*n* = 1,014)	Mean: 51.7 years (SD 10.2)	Male: 608 (60.0%)	NR	No prior treatments	No current treatments	Mean: 21.4 U/L (SD 7.6)	Mean: 1.86 log IU/mL (SD 0.72)	Positive: 0% Negative: 100%	No coinfections
Choi 2020 [25]	Full study cohort (*N* = 4,639)	Mean: 47.0 years (SD 10.9)	Male: 2,990 (64.5%)	Korean: 100%	No prior treatments	TDF: 1,574 (33.9%); ETV: 3,065 (66.1%)	Median: 93 U/mL (IQR 47–184)	Median: 6.7 log IU/mL (IQR 5.5–7.9)	Positive: 2,732 (64.2%)	No coinfections
Choi 2022a [26]	All treated patients (*N* = 2,073)	Mean: 42.1 years (SD 11.8)	Male: 1,306 (63.0%) Female: 767 (37.0%)	NR	No prior treatments	TDF: 792 (38.2%); ETV: 1,281 (61.8%)	Median: 131 IU/L (IQR 84–262)	Median: 8.0 log IU/mL (IQR 7.2–8.3)	Positive: 2,073 (100%)	No coinfections
Choi 2022b [27]	Full study cohort (*N* = 7,545)	Median: 44 years	Male: 61.60%	NR	No prior treatments	ETV or TDF: 100%	NR	NR	Positive: 100%	NR
Chun 2021 [28]	Individuals without cirrhosis at baseline (*n* = 7,602)	Mean: 48 years (SD 10)	Male: 4,665 (61.4%)	NR	No prior treatments	ETV or TDF: 100%	Median: 69 IU/L (IQR 40–125)	NR	NR	No coinfections
Dezanet 2021 [29]	Full study cohort (*N* = 169)	Median: 41.5 years (IQR 36.0-48.3)	Male: 83.4%	NR	LAM: 149 (88.2%)	TDF-based ART/TAF-based ART: 100%	NR	NR	Positive: 99 (58.6%)	HIV: 100%
Hosaka 2010 [30]	Individuals who developed HCC^a^ (*n* = 18)	Median: 52 years (range 35–75)	Male: 15 (83%)	NR	LAM: 100%	ADV + LAM: 100%	Median: 151 IU/L (range 61–576)	Median: 7.1 log copies/mL (range 4.4->7.6)	Positive: 8 (44%)	NR
	Individuals who did not develop HCC^a^ (*n* = 229)	Median: 45 years (range 26–75)	Male: 183 (80%)	NR	LAM: 100%	ADV + LAM: 100%	Median: 104 IU/L (range 13 − 1,563)	Median: 7.1 log copies/mL (range < 2.6->7.6)	Positive: 132 (58%)	NR
Hosaka 2013 [31]	ETV-treated individuals^a^ (*n* = 316)	Mean: 46 years (SD 12.1)	Male: 210/316 Female: 106/316	Japanese: 100%	No prior treatments	ETV: 100%	Median: 61 IU/L (IQR 39–109)	Median: 6.3 log copies/mL (IQR 5.2–7.9)	Positive: 135 (43%)	No coinfections
	LAM-treated individuals^a^ (*n* = 182)	Mean: 45 years (SD 10.7)	Male: 136/182 Female: 46/182	Japanese: 100%	No prior treatments	LAM: 100%	Median: 73 IU/L (IQR 42–121)	Median: 6.4 log copies/mL (IQR 5.0-7.4)	Positive: 71 (39%)	No coinfections
	Untreated individuals^a^ (*n* = 316)	Mean: 46 years (SD 13.5)	Male: 210/316 Female: 106/316	Japanese: 100%	No prior treatments	No current treatments	Median: 60 IU/L (IQR 28–144)	Median: 6.6 log copies/mL (IQR 4.5–7.8)	Positive: 133 (42%)	No coinfections
Hosaka 2022 [32]	Full study cohort (*N* = 180)	Mean: 51 years (SD 9.9)	Male: 111 (61.7%)	NR	NR	ETV: 100%	Median: 51 IU/L (IQR 31–98)	Median: 4.8 log IU/mL (IQR 3.5–5.6)	Negative: 100%	No coinfections
Ji 2022 [33]	ETV-treated (*n* = 500)	NR	NR	NR	NR	ETV: 100%	NR	NR	Positive: 100%	No coinfections
Kim 2015 [34]	Individuals without cirrhosis at baseline (*n* = 482)	Mean: 38.4 years (SD 11.8)	Male: 345 (72%)	White: 283 (59%) Asian: 148 (31%) Other: 51 (11%)	NR	TDF: 100%	Mean: 143 U/L (SD 113.1)	Mean: 7.7 log copies/mL (SD 1.5)	Positive: 199 (41%)	No coinfections
Kim 2017a [35]	Individuals without cirrhosis at baseline (*n* = 1,185)	Mean: 44 years (SD 11)	Male: 779 (65.7)	NR	No prior treatments	ETV: 100%	Median: 131 IU/mL (IQR 84–265)	NR	NR	No coinfections
Kim 2017b [36]	Full study cohort^a^ (*N* = 875)	Mean: 47.7 years (SD 10.7)	Male: 564 (64.5%)	Korean: 100%	No prior treatments	ETV: 100%	Median: 89 U/L (IQR 51–145)	Mean: 6.5 log IU/mL (SD 1.3)	Positive: 483 (55.2%)	No coinfections
Kim 2018 [37]	Non-cirrhotic, propensity-matched individuals receiving TDF treatment (*n* = 198)	Mean: 48 years (SD 11)	Male: 132 (66.7%)	NR	No prior treatments	TDF: 100%	Mean: 188.2 IU/mL (SD 280.9)	Mean: 6.4 log IU/mL (SD 1.6)	Positive: 152 (77.3%)	No coinfections
Ko 2019 [38]	Individuals who developed^a^ HCC (*n* = 66)	Median: 57.0 years (IQR Q3 − Q1 10.4)	Male: 54 (81.8%)	Asian: 100%	NA: 2 (3%)	ETV: 100%	Median: 58.5 U/L (IQR Q3 − Q1 48)	Median: 6.1 log IU/mL (IQR Q3 − Q1 2.8)	Positive: 14 (21.2%)	NR
	Individuals who did not develop HCC^a^ (*n* = 1,159)	Median: 49.4 years (IQR Q3 − Q1 16.1)	Male: 741 (63.9%)	Asian: 100%	NA: 76 (6.6%)	ETV: 100%	Median: 69 U/L (IQR Q3 − Q1 119)	Median: 6.5 log IU/mL (IQR Q3 − Q1 2.4)	Positive: 293 (25.3%)	NR
Kobashi 2011 [39]	Full study cohort (*N* = 256)	Median: 50 years (range 22–88)	Male: 179 (69.9%) Female: 77 (30.1%)	NR	No prior treatments	ETV: 129 (50.4%) LAM: 127 (49.6%)	Median: 87 IU/L (IQR 13 − 1,660)	Median: 7 log copies/mL (range 2.6–8.8)	Positive: 132 (51.6%) Negative: 124 (48.4%)	No coinfections
Kozuka 2019 [40]	Non-cirrhotic individuals receiving ETV treatment (*n* = 105)	Median: 43 years (range 23–78)	Male: 67 (63.8%)	Asian: 100%	IFN: 22 (21%)	ETV: 100%	Median: 119 U/L (range 22 − 2,605)	Median: 7.4 log copies/mL (range 3.1->9.1)	Positive: 52 (49.5%)	No coinfections
Lampertico 2007 [41]	Full study cohort^a^ (*N* = 145)	Median: 56 years (range 19–77)	Male: 122 (84%)	White: 100%	LAM: 100%	ADV + LAM: 100%	Median: 58 IU/L (range 13 − 2,870)	Median: 6 log copies/mL (range 3.5–9.3)	Negative: 124 (86%)	NR
Lee 2016 [42]	Full study cohort^a^ (*N* = 102)	Mean: 46.4 years (SD 11.2)	Male: 67 (66%) Female: 35 (34%)	NR	No prior treatments	ETV: 100%	Mean: 224.9 U/L (SD 440.8)	Median: 7,340,040.5 IU/mL (IQR 474,098.3–138,000,000)	Positive: 71 (70%)	NR
Lee 2019 [43]	Full study cohort^a^ (*N* = 462)	Mean: 51.1 years (SD 9.4)	Male: 250 (54.1%)	NR	No prior treatments	ETV: 231 (50.0%) LAM: 231 (50.0%)	Mean: 40.0 IU/L (SD 30.3)	Mean: 5.5 log IU/mL (SD 1.3)	Positive: 222 (48.1%)	No coinfections
Lee 2021 [44]	Propensity-score matched with TDF combination-therapy (*n* = 2,499)	Mean: 51.5 years (SD 8.7)	Male: 1,765 (70.6%)	Almost all patients had Korean ethnicity	ADV: 159 (6.4%); CLV: 202 (8.1%); ETV: 192 (7.7%); LAM: 1,900 (76.0%); telbivudine: 46 (1.8%)	TDF-based combination therapy: 100%	Median: 28 IU/mL (IQR 21–40)	NR	NR	No coinfections
	Propensity-score matched with TDF monotherapy (*n* = 2,499)	Mean: 50.7 years (SD 8.8)	Male: 1,712 (68.5%)	Almost all patients had Korean ethnicity	ADVr: 127 (5.1%); CLV: 308 (12.3%); ETV: 562 (22.5%); LAM: 1,426 (57.1%); LdT: 76 (3.0%)	TDF: 100%	Median: 27 IU/mL (IQR 20–41)	NR	NR	No coinfections
Lee 2022 [45]	Derivation cohort^a^ (*n* = 1,239)	Mean: 47.72 years (SD 10.95)	Male: 736 (59.4%)	NR	NR	TDF: 471 (38.3%); ETV: 760 (61.7%)	Median: 87 U/L (IQR 44–180)	Median: 1.8 × 10^6^ IU/mL (IQR 7.5 × 10^4^-3.26 × 10^7^)	Positive: 625 (51.6%)	NR
Li 2017 [46]	Full study cohort^a^ (*N* = 1,200)	Mean/median NR	Male: 816/1,200 Female: 384/1,200	NR	No prior treatments	ADV: 455 (37.92%); ETV: 425 (35.42%); LAM: 153 (12.75%); LAM + ADV: 167 (13.92%)	Mean/median NR	Mean/median NR	Positive: 565 (47.08%)	No coinfections
Lim 2014 [47]	Non-cirrhotic LAM-treated individuals, propensity score matched to ETV-treated cohort (*n* = 878)	Mean: 42.2 years (SD 10.6)	Male: 578 (65.8%)	NR	No prior treatments	LAM: 100%	Median: 172 IU/mL (IQR 96–321)	Mean: 7.68 log IU/mL (SD 1.08)	Positive: 656 (74.7%)	No coinfections
Lin 2019 [48]	Individuals who developed^a^ HCC (*n* = 18)	Mean: 52.8 years (SD 6.1)	Male: 15 (83.3%)	NR	NR	ETV: 5/18; LAM: 8/18; LdT: 5/18	Mean: 161.4 IU/L (SD 177.3)	Mean: 6.7 × 10^6^ IU/mL (SD 130.0)	Positive: 7/18 Negative: 11/18	No coinfections
	Individuals who did not develop HCC^a^ (*n* = 378)	Mean: 47.1 (SD 12.6)	Male: 285 (75.4%)	NR	NR	ETV: 173/378; LAM: 160/378; LdT: 45/378	Mean: 361.7 IU/L (SD 496.3)	Mean: 150.0 × 10^6^ IU/mL (SD 160.0)	Positive: 164/378 Negative: 214/378	No coinfections
Moon 2015 [49]	Full study cohort^a^ (*N* = 524)	Mean: 46.2 years (SD 12.2)	Male: 340 (64.9%)	NR	No prior treatments	CLV: 70 (13.4%); ETV: 161 (30.7%); LAM: 293 (55.9%); multi-drug treatment^b^	Mean: 157.6 IU/L (SD 195.0)	Mean: 6.4 log IU/mL (SD 1.2)	Positive: 360 (68.7%)	HCV: 4 (0.9%)
Nagata 2016 [50]	Non-cirrhotic HBeAg- individuals (*n* = 39)	Mean: 50.0 years (SD 11.8)	Male: 28 (72%) Female: 11 (28%)	NR	No prior treatments	ETV: 14 (36%); LAM: 25 (64%); multi-drug treatment^c^	Mean/median NR	Mean/median NR	Positive: 0% Negative: 100%	No coinfections
	Non-cirrhotic HBeAg+ individuals (*n* = 55)	Mean: 44.3 years (SD 14.5)	Male: 37 (67%) Female: 18 (33%)	NR	No prior treatments	ETV: 17 (31%); LAM: 38 (69%); multi-drug treatment^d^	Mean/median NR	Mean/median NR	Positive: 100% Negative: 0%	No coinfections
Niro 2013 [51]	Individuals without cirrhosis at baseline (*n* = 74)	Mean: 58 years (SD 11)	Male: 55 (74%)	All but 1 patient was Italian	NA: 38 (51%); IFN monotherapy: 14 (19%); IFN + NA therapy: 24 (32%)	NA: 100%	Mean: 243 [units NR] (SD 245)	Mean/median NR	NR	No coinfections
Oh 2020 [52]	Propensity-matched individuals receiving ETV^a^ (*n* = 516)	Mean: 49.2 years (SD 12.6)	Male: 319 (61.8%)	NR	No prior treatments	ETV: 100%	NR	Median: 6.4 log IU/mL (IQR 5.4–7.5)	Positive: 314 (60.9%)	No coinfections
	Propensity-matched individuals receiving TDF^a^ (*n* = 516)	Mean: 49.0 years (SD 9.4)	Male: 325 (63.0%)	NR	No prior treatments	TDF: 100%	NR	Median: 6.4 log IU/mL (IQR 5.4–7.5)	Positive: 311 (60.3%)	No coinfections
Orito 2015 [53]	Full study cohort^a^ (*N* = 602)	Median: 52 years (range 21–79)	Male: 381/602 Female: 221/602	NR	NR	ETV: 405/602; LAM: 56/602; multi-drug combination therapy^e^	Median: 69 IU/L (range 9 − 2,821)	Median: 6.8 log copies/mL (range 2.3–9.1)	Positive: 295/600 Negative: 305/600	No coinfections
PAGE-B Cohort [54]	Full study cohort^a^ (*N* = 1,427)	Mean: 52.1 years (SD 13.1)	Male: 992 (69.5%)	White: 100%	NA: 642 (45.0%); IFN: 358 (25.1%)	TDF: 609 (42.7%); ETV: 515 (36.1%); ETV + ADV: 10 (0.7%); ETV + TDF: 20 (1.4%); TDF + LAM: 268 (18.8%); TDF + LdT: 5 (0.3%)	Mean/median NR	Mean/median NR	Positive: 261 (18.4%)	No coinfections
Papatheodoridis 2015 [55]	Full study cohort^a^ (*N* = 1,666)	Mean: 53.7 years (SD 13.5)	Male: 1,194 (71.7%)	White: 100%	NA: 659 (39.6%); IFN: 418 (25.1%)	TDF: 606 (36.4%); ETV: 715 (42.9%); ETV + ADV: 14 (0.8%); ETV + TDF: 24 (1.4%); TDF + LAM: 305/1,666; TDF + LdT: 2/1,666	Mean/median NR	Mean/median NR	Positive: 257 (15.4%)	No coinfections
Papatheodoridis 2017 [56]	Non-cirrhotic individuals receiving ETV or TDF (*n* = 1,379)	Mean: 51 years (SD 14)	Male: 932 (68%)	White: 100%	NA: 524 (38%); IFN: 324 (24%)	TDF: 626 (45%); ETV: 577 (42%); ETV + ADV: 11 (1%); ETV + TDF: 27 (2%); TDF + LAM: 131/1,379; TDF + LdT: 7/1,379	Mean/median NR	Mean/median NR	Positive: 266 (19%)	No coinfections
Papatheodoridi 2022 [57]	Propensity score-matched individuals who discontinued NA therapy (*n* = 245)	Mean: 57 years (SD 12)	Male: 178 (73%)	White: 143 (58%) Asian: 102 (42%)	TDF: 108 (44.1%); ETV: 111 (45.3%); LAM: 21 (8.6%)	No current treatments	Median: 111 IU/L (IQR Q3 − Q1 67)	Median: 5.8 log IU/mL (IQR Q3 − Q1 2.2)	Negative: 100%	No coinfections
	Propensity score-matched individuals who continued NA therapy > 5 years (*n* = 245)	Mean: 58 years (SD 12)	Male: 178 (73%)	White: 143 (58%) Asian: 102 (42%)	NR	NA: 100%; TDF: 92 (37.6%); ETV: 133 (54.3%); LAM: 15 (6.1%)	Median: 144 IU/L (IQR Q3 − Q1 415)	Median: 5.6 log IU/mL (IQR Q3 − Q1 2.7)	Negative: 100%	No coinfections
Pellicelli 2014 [59]	Non-cirrhotic individuals receiving NA therapy ≥ 18 months (*n* = 193)	Mean: 52.8 years (SD 13)	Male: 135 (70%)	White: 100%	IFN: 83 (43%)	TDF: 14/193; ADV: 6/193; ETV: 93/193; LAM: 57/193; LdT: 4/193; LAM + ADV: 19/193; NA rescue therapy: 35 (18%)	Mean: 86 IU/L (SD 40)	Mean: 5.2 log IU/mL (SD 1.7)	Negative: 100%	No coinfections
RETRACT-B [60]	Full study cohort^a^ (*N* = 1,552)	Mean: 52.9 years (SD 11.2)	Male: 1,122 (72.3%)	White: 175 (11.3%) Black: 13 (0.8%) Asian: 1,359 (87.6%) Other: 5 (0.3%)	NA: 100%; TDF: 421 (27.1%); ETV: 981 (63.2%); IFN: 113 (8.6%)	No current treatments	Median: 0.6 xULN (range 0.4–0.8)	NR	Negative: 100%	No coinfections
Shim 2017 [61]	Non-cirrhotic individuals treated with ETV (*n* = 133)	Median: 49 years (IQR 44–55)	Male: 75 (56.4%)	Korean: 100%	No prior treatments	ETV: 100%	Median: 105 U/L (IQR 68–213)	Median: 6.81 log IU/mL (IQR 5.55–7.97)	Positive: 75 (56.4%)	No coinfections
Shin 2018 [62]	Full study cohort^a^ (*N* = 894)	Mean: 52.0 years (SD 11.3)	Male: 597 (66.8%)	NR	No prior treatments	ETV: 100%	Median: 89 IU/mL (IQR 46–153)	Mean: 6.39 log IU/mL (SD 1.41)	Positive: 537 (60.1%)	No coinfections
Sou 2020 [63]	Full study cohort^a^ (*N* = 1,397)	Median: 50 years (IQR Q3 − Q1 17)	Male: 1,018 (72.9%)	Asian: 100%	No prior treatments	ETV: 100%	Median: 104 U/L (IQR Q3 − Q1 205)	Median: 5.96 log IU/mL (IQR Q3 − Q1 2.35)	Positive: 491 (35.1%)	No coinfections
Tseng 2017 [64]	Untreated ERADICATE-B study cohort (*n* = 2,075)	Mean/median NR	Male: 1,222 (58.89%) Female: 853 (41.11%)	Asian: 100%	No prior treatments	No current treatments	Mean/median NR	Mean/median NR	Positive: 372 (17.93%) Negative: 1,703 (82.07%)	No coinfections
	NA-treated validation cohort (*n* = 532)	NR	NR	Asian: 100%	NR	NA: 100%	NR	Mean/median NR	NR	NR
Wang 2016 [65]	NA-experienced individuals (*n* = 105)	Mean: 48.9 years (SD 13.4)	Male: 83 (79%)	NR	NA: 100%	TDF: 100%	Mean: 208.2 IU/L (SD 336.6)	Mean: 6.2 log copies/mL (SD 1.9)	Positive: 51 (48.6%)	No coinfections
	NA-naïve individuals (*n* = 131)	Mean: 48.9 years (SD 12.9)	Male: 100 (76.3%)	NR	No prior treatments	TDF: 100%	Mean: 238.7 IU/L (SD 419.8)	Mean: 6.9 log copies/mL (SD 1.5)	Positive: 42 (32.1%)	No coinfections
Wong 2013 [66]	Non-cirrhotic individuals (*n* = 1,199)	Mean: 50 years (SD 12)	Male: 863 (72%)	NR	NA: 358 (30%); IFN: 28 (2%)	ETV: 100%	Mean: 156 IU/L (SD 339)	Mean: 5.0 log IU/mL (SD 2.2)	Positive: 358 (32%) Negative: 813 (68%)	No coinfections
Wu 2017 [67]	Individuals receiving TDF (*n* = 106)	Mean: 47.1 years (SD 12.1)	Male: 74 (70%)	NR	No prior treatments	TDF: 100%	Mean: 303 U/L (SD 372)	Mean: 7.35 log IU/mL (SD 0.70)	Positive: 50 (47.1%)	No coinfections
	Individuals receiving ETV (*n* = 313)	Mean: 47 years (SD 12.3)	Male: 230 (73%)	NR	No prior treatments	ETV: 100%	Mean: 316 U/L (SD 440)	Mean: 7.18 log IU/mL (SD 0.74)	Positive: 172 (55.0%)	No coinfections
Yamada 2015 [68]	Non-cirrhotic individuals receiving ETV (*n* = 404)	Mean: 51.3 years (SD 12.1)	Male: 233 (58%) Female: 171/404	NR	IFN: 44 (11%)	ETV: 100%	Mean: 156.1 IU/L (SD 210.8)	Median: 6.9 log copies/mL	Positive: 181 (45%) Negative: 219/404	No coinfections
Yang 2013 [69]	NA-naïve individuals^a^ (*n* = 323)	Mean: 48.0 years (SD 12.2)	Male: 256 (79.3%)	NR	IFN: 20 (6.2%)	ETV: 100%	Mean: 324.5 U/L (SD 476.4)	Mean: 6.4 log copies/mL (SD 1.5)	Positive: 118 (36.5%)	No coinfections
	NA-experienced individuals^a^ (*n* = 164)	Mean: 48.2 years (SD 11.2)	Male: 144 (87.8%)	NR	ADV: 45 (27.4%); LAM: 155 (94.5%); IFN: 15 (9.1%)	ETV: 100%	Mean: 277.9 U/L (SD 460.3)	Mean: 6.3 log copies/mL (SD 1.7)	Positive: 69 (42.1%)	No coinfections
Yang 2014 [70]	Individuals who received LAM prophylaxis for transplant (*n* = 26)	Mean: 41 years (SD 14.2)	Male: 16/26 Female: 10/26	NR	NR	LAM+immunosuppressive therapy: 100%	NR	Mean: 3.1 log IU/mL (SD 2.8)	Negative: 100%	No coinfections
	Individuals who received LAM post-transplant (*n* = 28)	Mean: 42.7 years (SD 10.5)	Male: 18/28 Female: 10/28	NR	NR	LAM+immunosuppressive therapy: 100%	NR	Mean: 2.6 log IU/mL (SD 2.1)	Negative: 100%	No coinfections
Yip 2020 [71]	ETV-treated individuals^a^ (*n* = 28,041)	Mean: 53.4 years (SD 13.0)	Male: 18.094 (64.5%)	Asian: 100%	No prior treatments	ETV: 100%	Median: 62.0 U/L (IQR 33.0-137.0)	Mean: 5.3 log IU/mL (SD 2.2)	Positive: 8,317 (29.7%)	No coinfections
	TDF-treated individuals^a^ (*n* = 1,309)	Mean: 43.2 years (SD 13.1)	Male: 591 (45.1%)	Asian: 100%	No prior treatments	TDF: 100%	Median: 43.0 U/L (IQR 25.0-103.0)	Mean: 4.9 log IU/mL (SD 2.7)	Positive: 721 (55.1%)	No coinfections
Yuen 2007 [72]	Non-cirrhotic LAM-treated individuals (*n* = 142)	Median: 33.9 years (range 20.2–54.4)	Male: 106/142 Female: 36/142	Asian: 100%	NA: 99/142	LAM: 100%	Median: 65 U/L (range 14–838)	Median: 8.7 log copies/mL (range 4.2–11.7)	Positive: 100%	No coinfections
	Non-cirrhotic untreated individuals (*n* = 124)	Median: 33.4 years (range 20.8–59)	Male: 90/124 Female: 34/124	Asian: 100%	NR	No current treatments	Median: 56.5 U/L (range 9-850)	Median: 6.8 log copies/mL (range 1.5–9.6)	Positive: 100%	No coinfections

*Abbreviations*: *ADV* adefovir, *ALT* alanine aminotransferase, *ART* antiretroviral therapy, *CHB* chronic hepatitis B, *CLV* clevudine, *ETV* entecavir, *HBeAg* hepatitis B e-antigen, *HBV* hepatitis B virus, *HCC* hepatocellular carcinoma, *HCV* hepatitis C virus, *HIV* human immunodeficiency virus, *IFN* interferon, *IQR* interquartile range, *IU* international unit, *LAM* lamivudine, *LdT* telbivudine, *NA* nucleos(t)ide analogue, *NR* not reported, *SD* standard deviation, *TAF* tenofovir alafenamide, *TDF* tenofovir disoproxil fumarate, *ULN* upper limit of normal

^a^Baseline characteristics were not reported separately for individuals without cirrhosis at baseline (study reports a sub analysis of HCC risk in non-cirrhotic individuals and therefore is eligible for SLR inclusion)

^b^251 out of 363 patients initially on initial LAM/LdT/CLV switched to ADV (*n* = 149), ETV (*n* = 90), and other NAs (*n* = 12). Of the 161 patients initially started on ETV, 2 patients switched to ETV + ADV and 2 patients switched to TDF

^c^17 out of 25 patients receiving LAM discontinued treatment. Of these 17 patients, 4 switched to ETV. 6 out of 14 patients receiving ETV discontinued treatment, of these, 2 patients restarted ETV after relapsing

^d^14 out of 38 patients receiving LAM discontinued treatment, of these, 2 patients started ETV after relapsing

^e^*n*=74 switched from LAM monotherapy to LAM + ADV; *n* = 67 switched from LAM to ETV

Regarding treatment status, five studies reported on untreated patients with CHB. The most common treatments received during follow-up were ETV (*n* = 39 studies), NA combinations (*n* = 26 studies), TDF (*n* = 19 studies), and LAM (*n* = 16 studies). HBeAg status varied across studies, with eight studies including only HBeAg-negative individuals and five including only HBeAg-positive individuals. In mixed populations (*n* = 37 studies), HBeAg positivity ranged from 15.4% to 77.3%. ALT levels were reported in 45 studies, with 15 studies reporting mean values ranging from 21.4 to 361.7 IU/L and 24 studies reporting median values ranging from 23.0 to 172.0 IU/L. HBV DNA was reported by 46 studies, showing mean levels ranging from 1.86 log IU/mL in untreated, HBeAg-negative individuals with inactive CHB [24], to 7.7 log IU/mL in individuals who were HBeAg-positive for at least 6 months prior to study enrollment [34]. Additionally, Moon 2015 included participants with hepatitis C virus (HCV) co-infection [49] and Dezanet 2021 included those with human immunodeficiency virus (HIV) co-infection [29]. 

### SLR outcomes

A summary of outcomes reported across included studies is shown in Table 3. Most studies assessed specific time points or mean/median follow-up durations of at least five years, and 24 studies assessed incidence beyond five years of follow-up. Incidence at specific timepoints less than five years was assessed by 17 studies. Incidence at specific timepoints at five years of follow-up or beyond was assessed by 24 studies.

**Table 3:** Summary of study outcomes

Study	Cohort	Cohort Description	*N*	Follow-up Duration	Assessment Timepoint	Cumulative Incidence		Incidence Rate	Measures of Association
						*n*	%		
Ando 2018 [22]	NA-treated virological responders	Patients with sustained disappearance of viremia, defined as continuous HBV DNA levels < 2.1 log copies/mL	105	Median: 4.8 years (range 1.0–13.1)^a^	1 year	NR	0	NR	NR
					3 years	NR	2.2	NR	NR
					5 years	4	5.3	NR	HR: 5.563 (95% CI: 1.438, 21.519), *p* = 0.013 (cirrhotic vs. non-cirrhotic)
Chiu 2020 [23]	All PSM TDF or ETV discontinuation patients	All PSM patients, all of whom were HBeAg- and discontinued ETV or TDF treatment per 2012 APASL guidelines; follow-up for HCC development may include re-treatment period for some patients	460	TDF: mean: 158.7 weeks (SD 10.8) ETV: mean: 159 weeks (SD 29.1)	60 months	13	3.5	NR	NR
Cho 2014 [24]	NA complete responders	Complete responders to NA therapy, defined as continuous virological response (HBV DNA < 2,000 IU/mL)	754	Mean: 37.6 months (SD 21.6)^a^	1 year	NR	2.0	NR	NR
					3 years	NR	4.7	NR	NR
					5 years	NR	7.2	NR	NR
	NA incomplete responders	Incomplete responders to NA therapy, defined as HBV DNA fluctuation ≥ 2,000 IU/mL during follow-up	179	Mean: 51.7 months (SD 19.8)^a^	1 year	NR	3.3	NR	NR
					3 years	NR	6.4	NR	NR
					5 years	NR	11.1	NR	NR
	HBeAg- untreated inactive CHB	HBeAg- patients with normal ALT and serum HBV DNA < 2,000 IU/mL throughout follow-up	884	Mean: 42.7 months (SD 22.4)^a^	5 years	NR	0.8	NR	NR
	HBeAg- NA complete responders	HBeAg- complete responders to NA therapy, defined as continuous virological response (HBV DNA < 2,000 IU/mL)	316	Mean: 37.9 months (SD 21.5)^a^	5 years	15	6.9	NR	NR
Choi 2020 [25]	ETV- or TDF-treated	NR	2,612	Median: 5.6 years (range 1.0–10.9)^a^	Annual (entire follow-up)	NR	0.6	NR	HR: 2.30 (95% CI: 1.77, 2.99), *p* < 0.001 (cirrhotic vs. non-cirrhotic)
Choi 2022a [26]	All ETV- or TDF-treated patients	All treated patients, all of whom were HBeAg+	2,073	Median: 5.7 years (IQR 3.6–8.1)	Entire follow-up	47	NR	0.39 per 100 person-years (95% CI NR)	NR
	ETV- or TDF-treated, HBV DNA ≥ 8.00 log IU/mL	HBeAg+ treated patients with HBV DNA ≥ 8.00 log IU/mL	1,108	Median: 5.8 years (IQR 3.5–8.2)	Entire follow-up	10	NR	0.15 per 100 person-years (95% CI: 0.08, 0.27)	HR: 1 (reference)
	ETV- or TDF-treated, HBV DNA 7.00–7.99 log IU/mL	HBeAg+ treated patients with HBV DNA 7.00–7.99 log IU/mL	521	Median: 5.5 years (IQR 3.7–8.3)	Entire follow-up	14	NR	0.46 per 100 person-years (95% CI: 0.26, 0.75)	HR: 2.48 (95% CI: 1.10, 5.62), *p* = 0.03 (treated patients with HBV DNA 7.00–7.99 log IU/mL vs. treated patients with HBV DNA ≥ 8.00 log IU/mL)
	ETV- or TDF-treated, HBV DNA 6.00–6.99 log IU/mL	HBeAg+ treated patients with HBV DNA 6.00–6.99 log IU/mL	274	Median: 5.5 years (IQR 3.5–7.6)	Entire follow-up	13	NR	0.84 per 100 person-years (95% CI: 0.47, 1.40)	HR: 3.69 (95% CI: 1.58, 8.59), *p* = 0.002 (treated patients with HBV DNA 6.00–6.99 log IU/mL vs. treated patients with HBV DNA ≥ 8.00 log IU/mL)
	ETV- or TDF-treated, HBV DNA 5.00–5.99 log IU/mL	HBeAg+ treated patients with HBV DNA 5.00–5.99 log IU/mL	170	Median: 5.0 years (IQR 3.5–7.0)	Entire follow-up	10	NR	1.14 per 100 person-years (95% CI: 0.58, 2.03)	HR: 6.10 (95% CI: 2.46, 15.11), *p* < 0.001 (treated patients with HBV DNA 5.00–5.99 log IU/mL vs. treated patients with HBV DNA ≥ 8.00 log IU/mL)
	PSM high viral load	PSM cohort of high viral load patients, ≥ 8.00 log IU/mL, PSM to medium viral load patients, 5.00-7.99 log IU/mL	930	Median: 6.0 years (IQR 3.7–8.3)	Entire follow-up	10	NR	0.18 per 100 person-years (95% CI: 0.09, 0.31)	HR: 1 (reference)
	PSM medium viral load	PSM cohort of medium viral load patients, 5.00-7.99 log IU/mL, PSM to high viral load patients, ≥ 8.00 log IU/mL	930	Median: 5.4 years (IQR 3.6–7.9)	Entire follow-up	35	NR	0.66 per 100 person-years (95% CI: 0.47, 0.91)	HR: 3.76 (95% CI: 1.86, 7.59), *p* < 0.001 (PSM medium viral load patients vs. PSM high viral load patients)
	PSM untreated, moderate viral load	Untreated patients with moderate viral load, 5.00-7.99 log IU/mL, PSM to treated patients with moderate viral load	947	Median: 7.8 years (IQR 4.9–11.9)^a^	Entire follow-up	NR	NR	1.52 per 100 person-years (95% CI: 1.28, 1.81)	HR: 1 (reference)
	PSM treated, moderate viral load	HBeAg+ treated patients with moderate viral load, 5.00-7.99 log IU/mL, PSM to untreated patients with moderate viral load	947	Median: 5.6 years (IQR 3.5–8.0)^a^	Entire follow-up	NR	NR	0.67 per 100 person-years (95% CI: 0.47, 0.91)	HR: 0.44 (95% CI: 0.30, 0.64), *p* < 0.001 (PSM treated patients with moderate viral load vs. PSM untreated patients with moderate viral load)
	PSM untreated, high viral load	Untreated patients with high viral load, ≥ 8.00 log IU/mL, PSM to treated patients with high viral load	969	Median: 7.8 years (IQR 4.9–11.9)^a^	Entire follow-up	NR	NR	0.26 per 100 person-years (95% CI: 0.16, 0.39)	HR: 1 (reference)
	PSM treated, high viral load	HBeAg+ treated patients with high viral load, ≥ 8.00 log IU/mL, PSM to untreated patients with high viral load	969	Median: 5.6 years (IQR 3.5–8.0)^a^	Entire follow-up	NR	NR	0.10 per 100 person-years (95% CI: 0.04, 0.22)	HR: 0.48 (95% CI: 0.19, 1.21), *p* = 0.12 (PSM treated patients with high viral load vs. PSM untreated patients with high viral load)
Choi 2022b [27]	ETV- or TDF-treated, HBV DNA ≥ 8.00 log IU/mL	All included patients were HBeAg+	2,929	Median: 4.28 years^a^	Entire follow-up	NR	0.58	0.10 per 100 person-years (95% CI: 0.05, 0.17)	HR: 1 (reference)
	ETV- or TDF-treated, HBV DNA ≥ 7.00 and < 8.00 log IU/mL	All included patients were HBeAg+	1,984	Median: 4.28 years^a^	Entire follow-up	NR	3.08	0.48 per 100 person-years (95% CI: 0.35, 0.65)	HR: 4.04 (95% CI: 1.68, 9.73), *p* = 0.0020 (HBV DNA ≥ 7.00 and < 8.00 log IU/mL vs. HBV DNA ≥ 8.00 log IU/mL)
	ETV- or TDF-treated, HBV DNA ≥ 6.00 and < 7.00 log IU/mL	All included patients were HBeAg+	1,561	Median: 4.28 years^a^	Entire follow-up	NR	8.04	1.38 per 100 person-years (95% CI: 1.11, 1.69)	HR: 8.05 (95% CI: 3.34, 19.35), *p* < 0.0001 (HBV DNA ≥ 6.00 and < 7.00 log IU/mL vs. HBV DNA ≥ 8.00 log IU/mL)
	ETV- or TDF-treated, HBV DNA ≥ 5.00 and < 6.00 log IU/mL	All included patients were HBeAg+	1,071	Median: 4.28 years^a^	Entire follow-up	NR	7.51	1.17 per 100 person-years (95% CI: 0.88, 1.53)	HR: 6.23 (95% CI: 3.10, 12.51), *p* < 0.0001 (HBV DNA ≥ 5.00 and < 6.00 log IU/mL vs. HBV DNA ≥ 8.00 log IU/mL)
Chun 2021 [28]	ETV- or TDF-treated, totally sedentary	Patients with no physical activity	1,437^b^	Median: 3.1 years (IQR 2.0–4.6)^a^	Entire follow-up	NR	NR	2.43 per 100 patient-years (95% CI NR)	HR: 1 (reference)
	ETV- or TDF-treated, < 500 MET-min/week	Patients with a low level of physical activity as quantified using metabolic equivalent (MET)	2,425^b^	Median: 3.1 years (IQR 2.0–4.6)^a^	Entire follow-up	NR	NR	1.84 per 100 patient-years (95% CI NR)	HR: 0.83 (95% CI: 0.65, 1.06), *p* = 0.13 (< 500 MET-min/week vs. totally sedentary)
	ETV- or TDF-treated, 500–1,000 MET-min/week	Patients with a moderate level of physical activity as quantified using metabolic equivalent (MET)	2,326^b^	Median: 3.1 years (IQR 2.0–4.6)^a^	Entire follow-up	NR	NR	1.67 per 100 patient-years (95% CI NR)	HR: 0.72 (95% CI: 0.56, 0.92), *p* = 0.009 (500–1,000 MET-min/week vs. totally sedentary)
	ETV- or TDF-treated, 1,000–1,499 MET-min/week	Patients with a moderate level of physical activity as quantified using metabolic equivalent (MET)	988^b^	Median: 3.1 years (IQR 2.0–4.6)^a^	Entire follow-up	NR	NR	1.56 per 100 patient-years (95% CI NR)	HR: 0.66 (95% CI: 0.47, 0.92), *p* = 0.02 (1,000–1,499 MET-min/week vs. totally sedentary)
	ETV- or TDF-treated, ≥ 1,500 MET-min/week	Patients with a high level of physical activity as quantified using metabolic equivalent (MET)	433^b^	Median: 3.1 years (IQR 2.0–4.6)^a^	Entire follow-up	NR	NR	1.61 per 100 patient-years (95% CI NR)	HR: 0.68 (95% CI: 0.44, 1.06), *p* = 0.09 (≥ 1,500 MET-min/week vs. totally sedentary)
Dezanet 2021 [29]	TDF-/TAF-based ART-treated, low fibrosis without progression	HIV+ patients receiving TDF-/TAF-based ART with sustained no-to-mild fibrosis (F0-F1)	50	Median: 7.4 years (IQR 3.1–12.9)	Entire follow-up	0	0.0	NR	NR
	TDF-/TAF-based ART-treated, low fibrosis with progression	HIV+ patients receiving TDF-/TAF-based ART with initially low level of fibrosis, but a slow, linear increase towards advanced fibrosis during treatment	38	Median: 8.5 years (IQR 3.1–13.0)	Entire follow-up	0	0.0	NR	NR
	TDF-/TAF-based ART-treated, moderate fibrosis with high fluctuation	HIV+ patients receiving TDF-/TAF-based ART with a heterogenous distribution of initial fibrosis levels (ranging from F0-F1 to F4) and equally heterogenous fluctuations in FibroTest scores during treatment	67	Median: 6.6 years (IQR 3.2–13.2)	Entire follow-up	2	3.0	NR	NR
Hosaka 2010 [30]	LAM + ADV-treated	Patients with LAM-resistant mutations	186	Median: 115 weeks (range 25–282)	1 year	NR	2	NR	NR
					3 years	NR	11	NR	NR
					5 years	NR	16	NR	HR: 1.42 (95% CI: 1.04, 1.96), *p* = 0.03 (cirrhotic vs. non-cirrhotic)
Hosaka 2013 [31]	PSM ETV-treated	Patients who received ETV, PSM to the control group	237^b^	Median: 3.3 years (IQR 2.3–4.3)^a^	2 years	NR	0	NR	NR
					3 years	NR	0	NR	NR
					4 years	NR	0.8	NR	NR
					5 years	NR	2.5	NR	HR: 0.37 (95% CI: 0.15, 0.91), *p* = 0.030 (PSM ETV vs. PSM untreated)
	PSM untreated	Untreated historical control group, PSM to the ETV group	231^b^	Median: 7.6 years (IQR 3.4–13.7)^a^	2 years	NR	1.0	NR	NR
					3 years	NR	1.6	NR	NR
					4 years	NR	2.2	NR	NR
					5 years	NR	3.6	NR	HR: 1 (reference)
	PSM LAM-treated	Patients who received LAM, PSM to the ETV group	133^b^	Median: 6.8 years (IQR 5.0–9.9)^a^	2 years	NR	3.2	NR	NR
					3 years	NR	3.2	NR	NR
					4 years	NR	3.2	NR	NR
					5 years	NR	4.9	NR	NR
Hosaka 2022 [32]	ETV-treated	All included patients were HBeAg-	132^b^	Median: 11 years^a^	5 years	9	NR	NR	HR: 4.24 (95% CI: 1.81, 9.91), *p* = 0.001 (preexisting cirrhosis vs. no preexisting cirrhosis)
Ji 2022 [33]	ETV-treated	Patients who received ETV + placebo	500	Median: 89 months^a^	Entire follow-up	43	8.6	NR	NR
Kim 2015 [34]	TDF-treated	Patients receiving open-label TDF	482	Median: 7.36 years (range 0.00–7.55)^a^	Cumulative incidence: annual (entire follow-up) Incidence rate: entire follow-up	8	0.28	28.4 per 10,000 person-years (95% CI NR)	NR
Kim 2017a [35]	ETV-treated virological responders	Patients with a virological response to ETV, defined as serum HBV DNA < 15 IU/mL at 1 year of therapy	850	Median: 5.7 years (IQR 4.8–7.0)	Entire follow-up	37	NR	0.74 per 100 patient-years (95% CI: 0.52, 1.02)	NR
Kim 2017b [36]	ETV-treated maintained virologic response	Patients with complete virologic response to ETV, defined as HBV DNA < 12 IU/mL (limit of detection), and persistently undetectable HBV DNA throughout follow-up	224	Median: 4.5 years (range 1.0–8.7)^a^	5 years	NR	4.0	NR	HR: 1 (reference)
	ETV-treated low-level viremia	Patients with HBV DNA ≥ 12 IU/mL but < 2,000 IU/mL persistently or intermittently during follow-up, whether or not they previously achieved complete virologic response on ETV	208	Median: 4.5 years (range 1.0–8.7)^a^	5 years	NR	6.9	NR	HR: 1.65 (95% CI: 0.65, 4.17), *p* = 0.29 (low-level viremia vs. maintained virologic response)
	All ETV-treated patients	All relevant patients, including the maintained virologic response and low-level viremia cohorts	432	Median: 4.5 years (range 1.0–8.7)^a^	Entire follow-up	NR	NR	NR	HR: 3.53 (95% CI: 2.11, 5.94), *p* < 0.001 (cirrhotic vs. non-cirrhotic)
Kim 2018 [37]	PSM TDF-treated	Patients were PSM to an ETV-treated group	198	Median: 33 months (IQR 21–46)^a^	Entire follow-up	0	0	0 per 100 person-years (95% CI NR)	NR
Ko 2019 [38]	ETV-treated	NR	1,033^b^	Median: 6.6 years (IQR 2.8)^a^	Entire follow-up	38	3.7	NR	NR
					2 years	7	NR	NR	NR
					3 years	14	NR	NR	NR
					4 years	24	NR	NR	NR
					5 years	29	NR	NR	NR
					6 years	32	NR	NR	NR
					7 years	36	NR	NR	NR
					8 years	36	NR	NR	NR
					9 years	38	NR	NR	NR
					10 years	38	NR	NR	NR
Kobashi 2011 [39]	ETV- or LAM-treated	NR	194	Median: 4.25 years (range 0.41–10.0)^a^	1 year	NR	1.6	NR	NR
					3 years	NR	3.5	NR	NR
					5 years	NR	3.5	NR	NR
					7 years	NR	7.1	NR	NR
					10 years	14	29.6	NR	NR
Kozuka 2019 [40]	ETV-treated	NR	105	Median: 4.4 years (range 1.0–10.7)^a^	3 years	NR	3.5	NR	NR
					5 years	NR	5.4	NR	NR
					7 years	NR	5.4	NR	HR: 4.789 (95% CI: 1.296, 17.689), *p* = 0.019 (cirrhotic vs. non-cirrhotic)
Lampertico 2007 [41]	ADV- and LAM-treated	All patients had LAM-resistant HBV	39	Median: 42 months (range 12–74)^a^	Entire follow-up	0	NR	NR	NR
Lee 2016 [42]	ETV-treated	NR	66	Median: 45.2 months (IQR 36.0–58.3)^a^	1 year	NR	0	NR	NR
					3 years	NR	1.90	NR	NR
					5 years	NR	1.90	NR	HR: 9.07 (95% CI: 1.09, 75.69), *p* = 0.042 (cirrhotic vs. non-cirrhotic)
Lee 2019 [43]	ETV- or LAM-treated	Patients had a baseline METAVIR score of F3	72	NR	5 years	5	8.9	NR	NR
Lee 2021 [44]	PSM TDF combination therapy	PSM patients who received TDF-containing combination therapy after switching from other NA treatments	2,030	Median: 45.3 months (IQR 39.0–48.6)^a^	Entire follow-up	58	NR	0.75 per 100 patient-years (95% CI NR)	HR: 1 (reference)
	PSM TDF monotherapy	PSM patients who received TDF monotherapy for at least 6 months after switching from other NA treatments	2,030	Median: 43.7 months (IQR 34.8–49.0)^a^	Entire follow-up	67	NR	0.87 per 100 patient-years (95% CI NR)	HR: 1.21 (95% CI: 0.85, 1.71), *p* = 0.3 (PSM TDF monotherapy vs. PSM TDF combination therapy)
Lee 2022 [45]	ETV- or TDF-treated	NR	787	Median: 63 months (IQR 37.6–98.2)^a^	Entire follow-up	29	NR	NR	HR: 2.974 (95% CI: 1.865, 4.743), *p* < 0.0001 (cirrhotic vs. non-cirrhotic)
Li 2017 [46]	NA-treated	Patients with < 9.7 kPa LSM were considered to be patients without cirrhosis	965	NR	1 year	NR	0.83	NR	NR
					2 years	NR	1.83	NR	NR
					3 years	82	2.75	NR	NR
Lim 2014 [47]	PSM LAM-treated	Patients treated with LAM 100 mg/day, PSM to an ETV-treated cohort (which was not extracted due to overlap with other studies included in this SLR)	878	Median: 8.7 years (IQR 6.5–11.5)^a^	Entire follow-up	40	NR	0.80 per 100 patient-years (95% CI NR)	HR: 1.26 (95% CI: 0.68, 2.34), *p* = 0.46 (PSM ETV vs. PSM LAM)
Lin 2019 [48]	NA-treated	Patients treated with LAM, ETV, or LdT who were followed up for more than 2 years	270	Mean: 5 years	Entire follow-up	5	NR	NR	NR
					2 years	2	NR	NR	HR: 4.107 (95% CI: 1.424, 11.843), *p* = 0.009 (cirrhotic vs. non-cirrhotic)
Moon 2015 [49]	NA-treated	NR	330	Median: 48 months (range 8–60)^a^	1 year	NR	0	NR	NR
					2 years	NR	0.30	NR	NR
					3 years	NR	0.30	NR	NR
					4 years	NR	0.30	NR	NR
					5 years	NR	2.10	NR	HR: 17.16 (95% CI: 4.67, 63.12), *p* < 0.001 (cirrhotic vs. non-cirrhotic)
Nagata 2016 [50]	All ETV- or LAM-treated patients	All patients who started treatment with LAM or ETV, including several who discontinued treatment if they satisfied APASL stopping criteria	94	HBeAg-: mean: 108 months (range 18–182) HBeAg+: mean: 111 months (range 16–178)	Entire follow-up	5	5.3	NR	NR
	ETV- or LAM-treated, HBeAg-	HBeAg- patients who started treatment with LAM or ETV, including several who discontinued treatment if they satisfied APASL stopping criteria	39	Mean: 108 months (range 18–182)	Annual over entire follow-up	NR	0.6	NR	NR
	ETV- or LAM-treated, HBeAg+	HBeAg+ patients who started treatment with LAM or ETV, including several who discontinued treatment if they satisfied APASL stopping criteria	55	Mean: 111 months (range 16–178)	Annual over entire follow-up	NR	0.6	NR	NR
Niro 2013 [51]	Continuous NA therapy	NR	74	Mean: 6 years (SD 3)^a^	Entire follow-up	1	1	NR	NR
Oh 2020 [52]	PSM ETV-treated	NR	278	Median: 4.7 years (IQR 4.3–5.3)^a^	Entire follow-up	3	NR	NR	HR: 1 (reference)
	PSM TDF-treated	NR	278	Median: 4.8 years (IQR 3.9–5.4)^a^	Entire follow-up	4	NR	NR	HR: 1.41 (95% CI: 0.32, 6.32), *p* = 0.651 (PSM TDF vs. PSM ETV)
Orito 2015 [53]	All NA-treated patients	All relevant patients	492	Median: 90 months (range 12–204)	Annual over entire follow-up	13	0.34	NR	NR
	NA-treated, HBV DNA+	Patients who did not lose HBV DNA positivity during follow-up	188	Median: 90 months (range 12–204)^a^	Entire follow-up	15	8.0	NR	NR
	NA-treated, HBV DNA-	Patients who became HBV DNA- during follow-up	230	Median: 90 months (range 12–204)^a^	Entire follow-up	13	5.7	NR	NR
	NA-treated, HBeAg+ during therapy	Patients who were HBeAg+ during follow-up	99	Median: 90 months (range 12–204)^a^	Entire follow-up	4	4.0	NR	NR
	NA-treated, HBeAg seroconversion	Patients who experienced HBeAg seroconversion during follow-up	39	Median: 90 months (range 12–204)^a^	Entire follow-up	2	5.1	NR	NR
	NA-treated, HBsAg+ during therapy	Patients who were HBsAg+ during follow-up	479	Median: 90 months (range 12–204)^a^	Entire follow-up	13	2.7	NR	NR
	NA-treated, HBsAg seroclearance	Patients who experienced HBsAg seroclearance during follow-up	13	Median: 90 months (range 12–204)^a^	Entire follow-up	0	0.0	NR	NR
PAGE-B Cohort [54]	All NA-treated, baseline non-cirrhotic patients	Patients who did not develop HCC within the first 5 years of treatment	1,023	Median: 8.4 years^a^	Years 5–12	12	1.2	NR	HR: 4.81 (95% CI: 2.36, 9.79), *p* < 0.001 (cirrhotic vs. non-cirrhotic)
					6 years	NR	NR	0.5 per 100 patient-years (95% CI NR)	NR
					8 years	NR	NR	1.2 per 100 patient-years (95% CI NR)	NR
					10 years	NR	NR	1.7 per 100 patient-years (95% CI NR)	NR
	NA-treated, non-cirrhotic at baseline and at year 5	Patients who did not develop HCC within the first 5 years of treatment and had LSM < 12 kPa at year 5	870	Median: 8.4 years^a^	Years 5–12	9	NR	NR	NR
					6 years	NR	NR	0.5 per 100 patient-years (95% CI NR)	NR
					8 years	NR	NR	1.1 per 100 patient-years (95% CI NR)	NR
					10 years	NR	NR	1.3 per 100 patient-years (95% CI NR)	NR
	NA-treated, non-cirrhotic at baseline, cirrhotic at year 5	Patients who did not develop HCC within the first 5 years of treatment and had LSM ≥ 12 kPa at year 5	3	Median: 8.4 years^a^	Years 5–12	1	NR	NR	NR
Papatheodoridis 2015 [55]	ETV- and/or TDF-treated	NR	1,085	Median: 38.8 months (IQR 23.4–49.9)^a^	1 year	NR	0.6	NR	NR
					3 years	NR	1.4	NR	NR
					5 years	NR	3.7	NR	HR: 4.50 (95% CI: 2.55, 7.96), *p* < 0.001 (compensated cirrhotic vs. non-cirrhotic)
Papatheodoridis 2017 [56]	ETV- and/or TDF-treated	NR	1,379	Median: 72 months (IQR 35)^a^	5 years	NR	NR	0.49 per 100 patient-years (95% CI NR)	NR
	ETV- and/or TDF-treated	Patients analyzed after excluding person-time accrued and HCC cases diagnosed within the first year of follow-up	NR	Median: 72 months (IQR 35)^a^	5 years	NR	NR	0.51 per 100 patient-years (95% CI NR)	NR
Papatheodoridi 2022 [57]	PSM NA discontinuation	HBeAg- patients from a Caucasian cohort who discontinued NA treatment within 5 years and an Asian cohort who discontinued treatment within 3 years without developing HCC	245	Median: 47 months (IQR 34)^a^	1 year	7	0.9	NR	NR
					3 years	7	1.9	NR	NR
					5 years	7	5.1	NR	HR: 1.49 (95% CI: 0.44, 5.12), *p* = 0.523 (PSM NA discontinuation vs. PSM NA continuation)
	PSM NA continuation	HBeAg- patients from a Caucasian cohort who continued NA treatment beyond 5 years and an Asian cohort who continued treatment beyond 3 years without developing HCC	245	Median: 40 months (IQR 32)^a^	1 year	7	0.4	NR	NR
					3 years	7	2.5	NR	NR
					5 years	7	4.9	NR	HR: 1 (reference)
	NA discontinuation, with low virological relapse	Non-PSM HBeAg- patients with low virological relapse, defined as HBV DNA > 2,000 IU/mL	228	Median: 47 months (IQR 34)^a^	Entire follow-up	5	2.2	NR	HR: 1.64 (95% CI: 0.18, 14.78), *p* = 0.659 (NA discontinuation with vs. without low virological relapse)
	NA discontinuation, without low virological relapse	Non-PSM HBeAg- patients without low virological relapse, defined as HBV DNA > 2,000 IU/mL	97	Median: 47 months (IQR 34)^a^	Entire follow-up	2	2.1	NR	HR: 1 (reference)
	NA discontinuation, with high virological relapse	Non-PSM HBeAg- patients with high virological relapse, defined as HBV DNA > 20,000 IU/mL	172	Median: 47 months (IQR 34)^a^	Entire follow-up	4	2.3	NR	HR: 1.45 (95% CI: 0.24, 8.71), *p* = 0.688 (NA discontinuation with vs. without high virological relapse)
	NA discontinuation, without high virological relapse	Non-PSM HBeAg- patients without high virological relapse, defined as HBV DNA > 20,000 IU/mL	153	Median: 47 months (IQR 34)^a^	Entire follow-up	3	2.0	NR	HR: 1 (reference)
	NA discontinuation, with HBsAg seroclearance	Non-PSM HBeAg- patients with HBsAg seroclearance, which was assessed prior to HCC diagnosis	60	Median: 47 months (IQR 34)^a^	Entire follow-up	0	0.0	NR	HR: 2.61 (95% CI: 0.43, 15.63), *p* = 0.295 (NA discontinuation with vs. without HBsAg seroclearance)
	NA discontinuation, without HBsAg seroclearance	Non-PSM HBeAg- patients without HBsAg seroclearance, which was assessed prior to HCC diagnosis	265	Median: 47 months (IQR 34)^a^	Entire follow-up	7	2.6	NR	HR: 1 (reference)
Pellicelli 2014 [59]	LAM-treated	All patients were HBeAg- and had HBV genotype D	57	Mean: 69 months (SD 30)^a^	Entire follow-up	NR	NR	0.001 per 100 person-years (95% CI NR)	OR: 7.9 (95% CI: 0.74, 85.00), *p* = 0.08 (cirrhotic vs. non-cirrhotic)
	NA-treated	All patients were HBeAg- and had HBV genotype D	193	Mean: 69 months (SD 30)	Entire follow-up	2	1	NR	OR: 7.6 (95% CI: 1.37, 42.20), *p* = 0.02 (cirrhotic vs. non-cirrhotic)
					10 years	2	0.5	NR	NR
RETRACT-B [60]	NA discontinuation	All patients were HBeAg-, virally suppressed, and met their study center’s criteria for stopping NA therapy	1,368	Median: 18.4 years (range 7.9–39.4)^a^	Entire follow-up	10	0.7	NR	NR
Shim 2017 [61]	ETV-treated	NR	133	Median: 3.6 years (IQR 2.0–5.1)^a^	Entire follow-up	2	NR	4.0 per 1,000 patient-years (95% CI NR)	HR: 6.42 (95% CI: 1.42, 28.95), *p* = 0.016 (stage 1 cirrhotic vs. non-cirrhotic) HR: 24.89 (95% CI: 5.77, 107.30), *p* < 0.001 (stage 2 cirrhotic vs. non-cirrhotic) HR: 17.01 (95% CI: 3.86, 74.90), *p* < 0.001 (stage 3 cirrhotic vs. non-cirrhotic)
Shin 2018 [62]	ETV-treated	NR	454	Median: 5.4 years (IQR 3.0–7.3)^a^	Entire follow-up	7	1.5	NR	NR
					5 years	NR	1.5	NR	NR
					7 years	7	2.5	NR	NR
					Annual (7 years)	NR	0.4	NR	NR
	ETV-treated, with good adherence	Patients with ETV adherence ≥ 90%	320	Median: 5.4 years (IQR 3.0–7.3)^a^	Entire follow-up	5	1.6	NR	HR: 1.002 (95% CI: 0.978, 1.027), *p* = 0.836 (good vs. poor adherence)
					5 years	NR	1.7	NR	NR
					7 years	5	1.7	NR	NR
					Annual (7 years)	NR	0.2	NR	NR
	ETV-treated, with poor adherence	Patients with ETV adherence < 90%	134	Median: 5.4 years (IQR 3.0–7.3)^a^	Entire follow-up	2	1.5	NR	HR: 1 (reference)
					5 years	NR	1.5	NR	NR
					7 years	2	4.9	NR	NR
					Annual (7 years)	NR	0.7	NR	NR
Sou 2020 [63]	ETV-treated	NR	890	NR	5 years	NR	2.5	NR	NR
					Annual (0 to < 5 years)	NR	0.63	NR	NR
					10 years	NR	3.2	NR	NR
			340	NR	Annual (5–10 years)	NR	0.14	NR	NR
Tseng 2017 [64]	All ERADICATE-B untreated patients	All patients in the retrospective, untreated ERADICATE-B cohort	2,075	Median: 15.38 years (range 2.51–27.72)	Entire follow-up	137	NR	0.41 per 100 person-years (95% CI NR)	NR
	ERADICATE-B untreated patients with 15-year follow-up	Retrospective, untreated ERADICATE-B cohort patients who were followed-up for 15 years	1,241	Median: 15.38 years (range 2.51–27.72)^a^	15 years	105	NR	NR	NR
	ERADICATE-B untreated inactive carriers	Retrospective, untreated ERADICATE-B cohort patients who met the following criteria: HBeAg-, HBV DNA < 2,000 IU/mL, HBsAg < 1,000 IU/mL, and ALT < 40 U/L	408	Median: 15.38 years (range 2.51–27.72)^a^	Annual (15 years)	NR	0.06	NR	NR
	NA-treated validation cohort	Patients in the validation cohort received long-term NA therapy	532	Median: 6.58 years (range 1.28–11.88)	Entire follow-up	10	NR	0.27 per 100 person-years (95% CI NR)	NR
Wang 2016 [65]	TDF-treated	NR	141	Median: 154 weeks (range 52–196)	36 months	0	0	NR	NR
Wong 2013 [66]	ETV-treated	NR	1,199	Mean: 43 months (SD 13)	3 years	NR	1.8	NR	NR
					5 years	21	2.1	NR	HR: 3.2 (95% CI: 1.5, 6.4), *p* = 0.002 (cirrhotic vs. non-cirrhotic)
Wu 2017 [67]	ETV- or TDF-treated	NR	296	ETV-treated: mean: 49 months (SD 19.1)^a^ TDF-treated: mean: 37.9 months (SD 7.2)^a^	Entire follow-up	3	NR	NR	HR: 6.22 (95% CI: 1.81, 21.37), *p* = 0.004 (cirrhotic vs. non-cirrhotic)
Yamada 2015 [68]	ETV-treated	NR	404	Mean: 49.2 months (range 14–109)	3 years	16	2.7	NR	NR
					5 years	16	5.3	NR	HR: 4.765 (95% CI: 2.415, 9.404), *p* < 0.001 (cirrhotic vs. non-cirrhotic)
	ETV-treated, with virological response at 24 weeks	Patients with a virological response, defined by serum HBV DNA levels that were continuously < 2.6 log copies/mL	275^b^	Mean: 49.2 months (range 14–109)^a^	3 years	NR	3.5	NR	NR
					5 years	NR	7.2	NR	NR
	ETV-treated, without virological response at 24 weeks	Patients without a virological response, defined by serum HBV DNA levels that were continuously ≥ 2.6 log copies/mL	129^b^	Mean: 49.2 months (range 14–109)^a^	3 years	NR	1.0	NR	NR
					5 years	NR	1.0	NR	NR
Yang 2013 [69]	ETV-treated	NR	314	Median: 36 months (range 12–73)^a^	1 year	NR	0	NR	NR
					2 years	NR	0.7	NR	NR
					3 years	NR	1.9	NR	NR
					4 years	NR	3.80	NR	HR: 7.10 (95% CI: 2.68, 18.83), *p* < 0.001 (cirrhotic vs. non-cirrhotic)
Yang 2014 [70]	LAM prophylaxis	Renal transplant recipients who were HBeAg- and received LAM prophylaxis	26	Mean: 6.7 years (SD 2.4)	5 years	0	0	NR	NR
					10 years	0	0	NR	NR
	LAM post-transplant	Renal transplant recipients who were HBeAg- and received LAM later in the post-transplant period for HBV reactivation	28	Mean: 9.4 years (SD 4.7)	5 years	0	0	NR	NR
					10 years	1	4.2	NR	NR
Yip 2020 [71]	TDF-treated	NR	1,271	Median: 2.8 years (IQR 1.4–4.5)^a^	Entire follow-up	2	0.2	NR	NR
	ETV-treated	NR	24,219	Median: 3.7 years (IQR 1.7–5.0)^a^	Entire follow-up	646	2.7	NR	NR
Yuen 2007 [72]	LAM-treated	All patients were HBeAg+	142	Median: 89.9 months (range 26.5–128.3)	Entire follow-up	1	NR	NR	NR
	Untreated	All patients were HBeAg+	124	Median: 107.8 months (range 30.9–127.3)	Entire follow-up	3	NR	NR	NR

*Abbreviations*: *ADV* adefovir, *ALT* alanine transaminase, *APASL* Asian-Pacific Association for the Study of the Liver, *ART* antiretroviral therapy, *CI* confidence interval, *DNA* deoxyribonucleic acid, *ETV* entecavir, *HBeAg* hepatitis B e-antigen, *HR* hazard ratio, *HBsAg* hepatitis B surface antigen, *HBV* hepatitis B virus, *HCC* hepatocellular carcinoma, *HIV* human immunodeficiency virus, *IQR* interquartile range, *LAM* lamivudine, *LdT* telbivudine, *LSM* liver stiffness measurement, *MET* metabolic equivalent, *N* cohort size (denominator), *n* cases of HCC (numerator), *NA* nucleo(s/t)ide analog, *NR* not reported, *OR* odds ratio, *PSM* propensity score-matched, *SD* standard deviation, *TAF* tenofovir alafenamide, *TDF* tenofovir disoproxil

^a^Follow-up durations were not reported separately for the cohort of interest and may include patients for whom outcomes were not extracted

^b^Cohort sample size calculated with information provided

Cumulative incidence was reported by all but two studies, seven of which reported the annual cumulative incidence ranging from 0.06% in untreated, HBeAg negative inactive carriers [64] to 0.72% in ETV-treated individuals with medication adherence rates of < 90% [62]. The cumulative incidence of HCC in both treated and untreated cohorts was also reported across specific follow-up periods. Over a one-year follow-up (reported by 10 studies), the incidence ranged from 0 to 3.3%. This range increased over three years (reported by 16 studies), reaching 0 to 11.0%, and remained similar at five years (reported by 21 studies), with values from 0 to 11.1%. By ten years (reported by six studies), the cumulative incidence expanded further, ranging from 0% to 29.6%.

The 13 studies reporting incidence rates assessed a total of 38 cohorts or timepoints. The sum of person-years across studies ranged from 500.6 to 33,249.7. Reported incidence rates ranged from 0 per 100 person-years in TDF-treated individuals propensity score matched to an ETV-treated group [37], to 2.43 per 100 person-years in ETV- or TDF-treated individuals who were completely sedentary in a study examining the association between physical activity and risk of HCC in patients with CHB [28]. Overall, of the 38 cohorts or timepoints assessed, 15 of the reported incidence rates were ≤ 0.5 per 100 person-years, 10 were > 0.5 and ≤ 1 per 100 person-years, and 13 were > 1 per 100 person-years. Hazard ratios (HR) assessing the risk of HCC development were reported by 26 studies, and odds ratios (OR) were reported by one study. Among these, 18 studies compared individuals with baseline cirrhosis to those without baseline cirrhosis, while seven studies compared treatment groups, such as the type of NA treatment received, treatment versus no treatment, treatment discontinuation, and treatment adherence. Additionally, four studies compared cohorts with varying HBV DNA levels or virological responses to treatment.

Surveillance and diagnostic methodologies were reported by all studies, summarized in Table S9. Hosaka 2010 reported two sets of methodologies across publications [30, 73], which are considered separately in this section. Therefore, of 51 study methodologies, 37 reported HCC surveillance frequency, 33 reported surveillance details, 21 reported diagnostic guidelines, and 35 reported diagnostic details. Generally, patients were screened for HCC approximately every six months; 14 study methodologies screened patients every six months, 12 every three to six months, and three every six to 12 months. Two study methodologies reported more frequent screening, with one cohort of Hosaka 2010 screening patients monthly [30], and Ji 2022 screening patients at least every 12 weeks [33]. Four study methodologies reported less frequent screening, with the PAGE-B cohort and Papatheodoridis 2017 reporting screening at least every 12 months [54, 56], Papatheodoridis 2015 reporting approximately annual screening for all patients [55], and Wong 2013 reporting screening every one to two years [66]. 

All studies that reported surveillance methodology also utilized imaging, most frequently ultrasonography and/or alpha-fetoprotein (AFP) measurement, along with other tumor markers in some cases. Diagnostic guidelines reported were AASLD (*n* = 11), EASL (*n* = 5), AASLD and EASL (*n* = 2), Korean Liver Cancer Study Group (*n* = 2), and Liver Cancer Study Group of Japan (*n* = 1). Three additional study methodologies reported following certain diagnostic guidelines, although the specific guidelines were not reported. HCC was diagnosed by imaging, biopsy/histology, and/or AFP measurement, as reported by 35 studies. Two additional database studies did not report diagnostic criteria but reported that International Classification of Diseases (ICD) codes were used to identify patients who developed HCC. Of the study methodologies that did report specific diagnostic criteria, nine diagnosed HCC based on imaging alone, while others utilized a combination of diagnostic techniques.

### Feasibility assessment and meta-analysis

Across studies, substantial differences were observed in both study and patient characteristics. The feasibility assessment determined that analyzing all studies identified in the SLR to assess rates of HCC incidence across populations with average covariate values indicating earlier treatment initiation (i.e., younger age or higher baseline viral load) would be of limited robustness due to the extent of heterogeneity and aggregate nature of the data. Additionally, study populations differed notably in terms of treatment status, in that some were only treated before study follow-up. Four studies identified in the SLR (Choi 2022a [26], Yuen 2007 [72], Tseng 2017 [64], and Hosaka 2013[31]) included both treated and untreated populations and reported HCC incidence for each. These studies were therefore deemed feasible for inclusion in a direct comparison meta-analysis which allowed for evaluation of HCC risk among populations within the same study, ensuring that differences in outcomes can be attributed more reliably to antiviral treatment effects.

Following the conclusion of the SLR, a targeted literature review update performed in December 2024 identified a more recent publication by Choi et al. in a cohort which overlapped with the original study selected for the meta-analysis [74]. This new publication included a larger sample size, including both HBeAg-positive and -negative individuals, whereas the originally identified study was limited to those who were HBeAg-positive. Therefore, data from the newer publication were used in place of the prior one for the meta-analysis. A summary of this study is provided in Table 4.

**Table 4:** Summary of Choi 2024 included in the meta-analysis

Study	Study Design	Study Country	Sample Size	Treatments Assessed	Cohort Description	Definition of cirrhosis status	Outcomes
Choi 2024 [74]	Retrospective cohort	South Korea	4,693	ETV or TDF	HBeAg-positive and -negative individuals who were treatment naïve at baseline	Cirrhosis was defined as the presence of any of the following characteristics: coarse liver echotexture and nodular liver surface on ultrasonography; clinical features of portal hypertension (e.g., ascites, splenomegaly and varices); and platelet count < 100 × 10^3^/µL	193 patients developed HCC (0.53 per 100 person-years; 95% CI NR)

*CI* Confidence interval, *ETV* Entecavir, *HBeAg* Hepatitis B e-antigen, *HCC* Hepatocellular carcinoma, *NR* Notreported, *TDF* tenofovir disoproxil

**Fig. 1:**
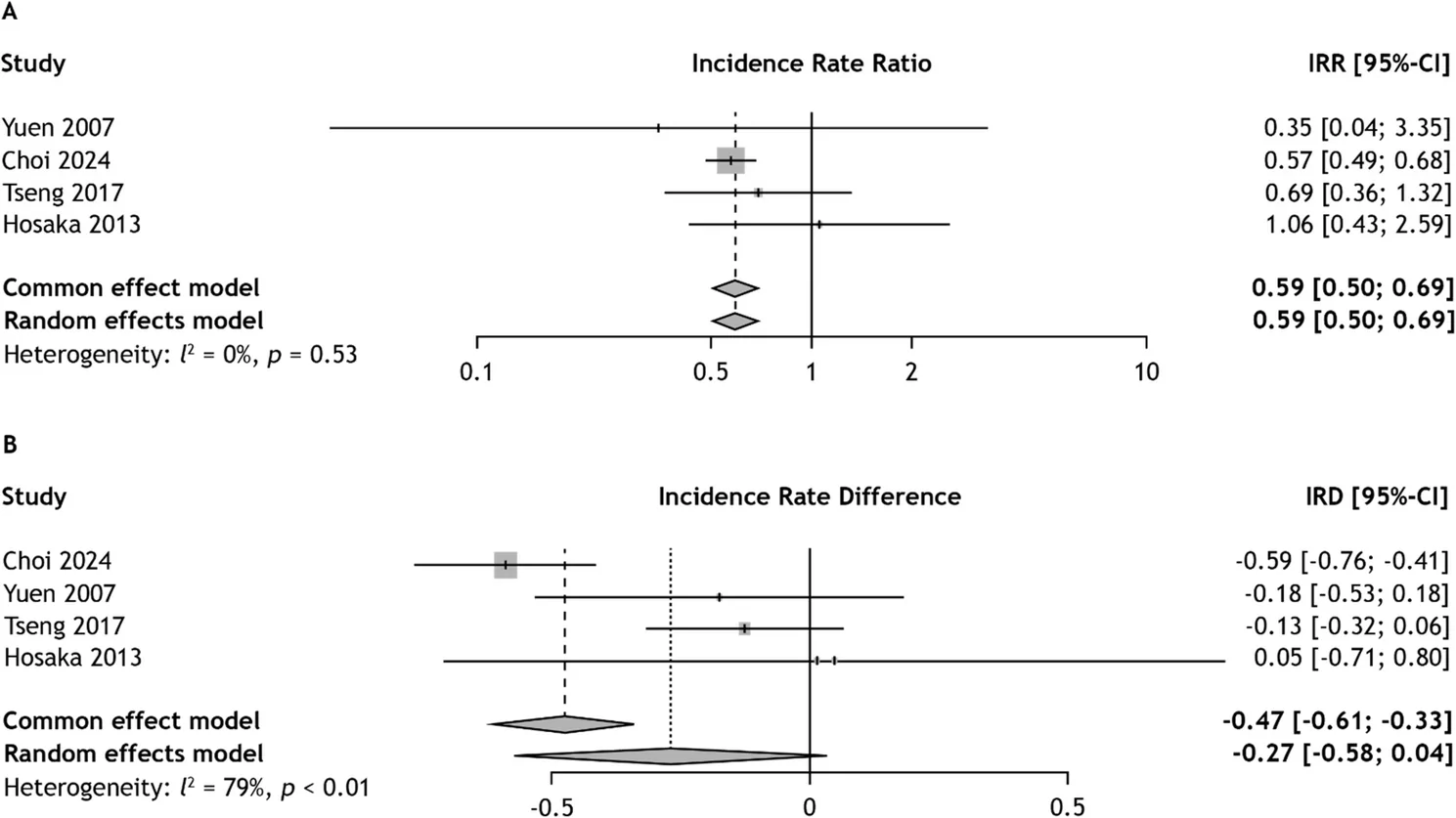
Unadjusted comparative meta-analysis of HCC incidence in treated versus untreated populations, IRR (Panel **A**) and IRD (Panel **B**). Abbreviations: CI, confidence interval; IRD, incidence rate difference; IRR, incidence rate ratio

All studies included in the direct comparison meta-analysis were retrospective or prospective cohorts conducted in Asia. Average duration of follow-up ranged from 3.3 to 15.4 years. Notable differences were observed in patient characteristics between the treated and untreated populations of Tseng 2017 and Yuen 2007, which did not use propensity score matching or similar methods. From the random effects model, an IRR of 0.59 (95% CI: 0.50, 0.69) was found, indicating that treated populations had 0.59 times the rate of HCC compared to untreated populations, shown in Panel A of Fig. 1. An IRD of -0.27 (95% CI: -0.58, 0.04) was found, indicating 27 (-4, 58) excess cases of HCC per 100 person-years of follow-up among untreated individuals attributable to treatment status (shown in Panel B of Fig. 1).

### Sensitivity analysis

**Fig. 2:**
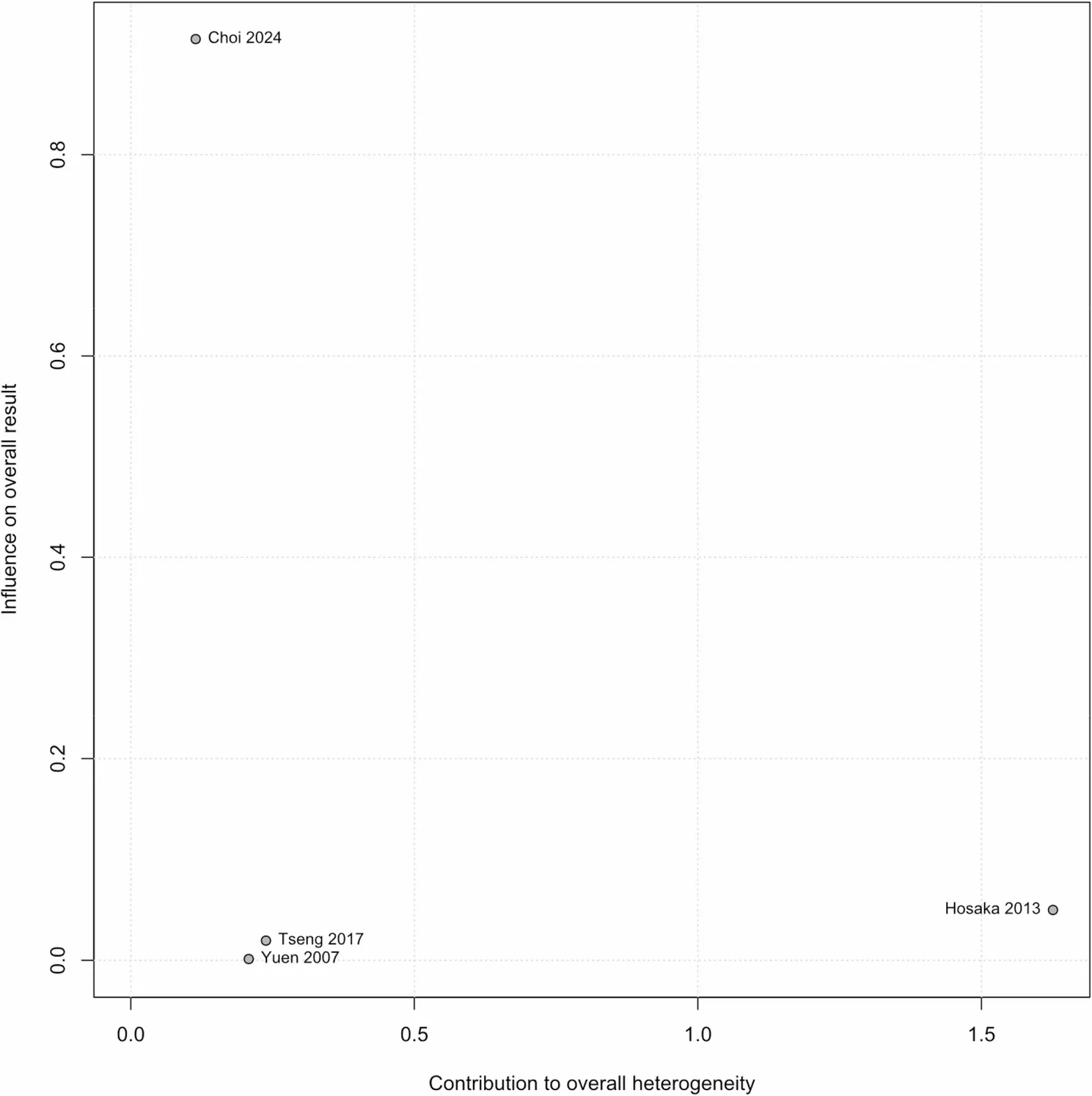
Sensitivity analysis: Baujat plot of the unadjusted comparative meta-analysis

**Fig. 3:**
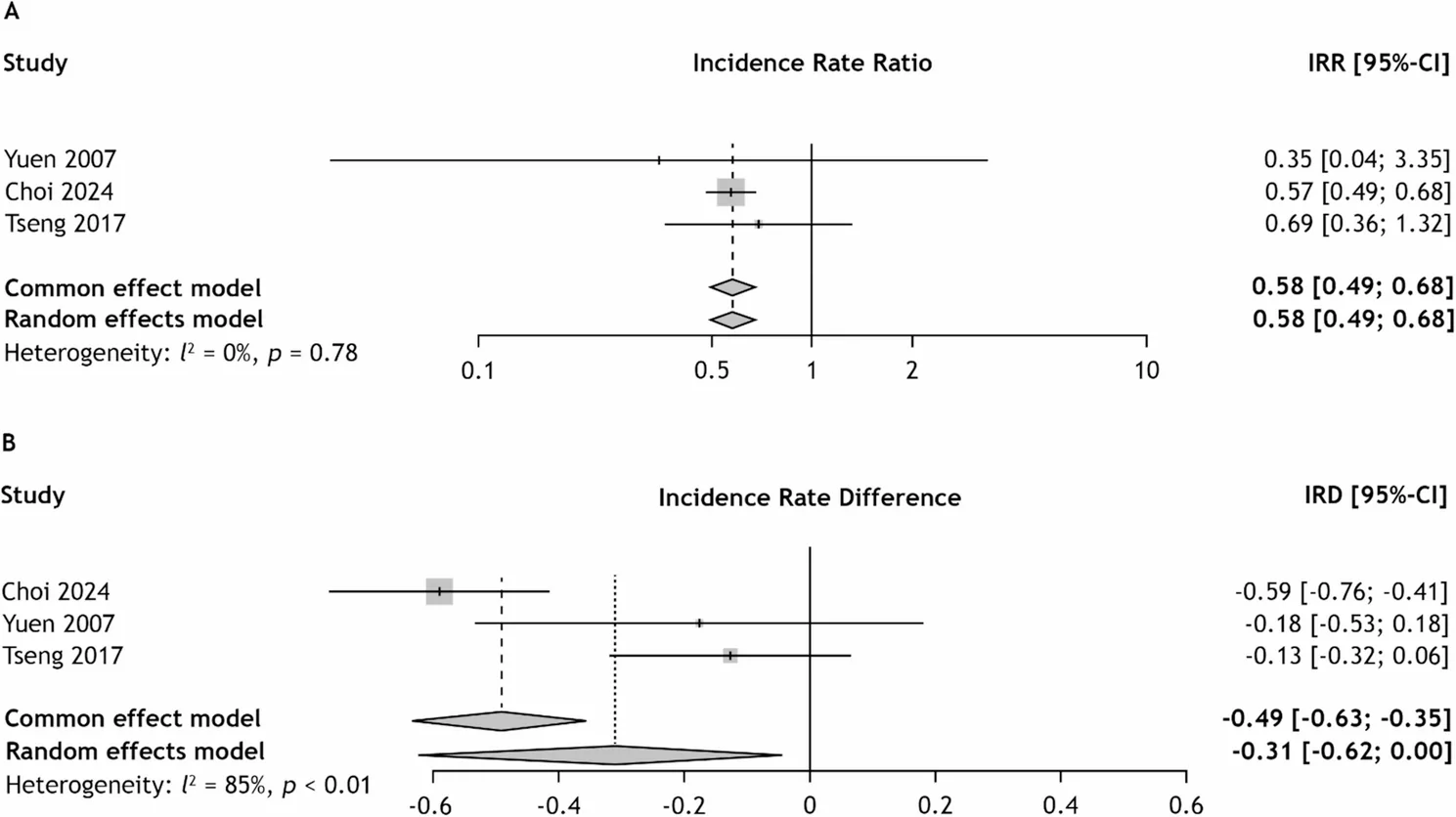
Sensitivity analysis of unadjusted comparative meta-analysis of HCC incidence in treated versus untreated populations, IRR (Panel **A**) and IRD (Panel **B**). Abbreviations: CI, confidence interval; IRD, incidence rate difference; IRR, incidence rate ratio

A Baujat plot constructed to explore sources of heterogeneity in the meta-analysis identified Hosaka 2013 as a potential outlier based on its contribution to the pooled treatment effect and to overall heterogeneity, shown in Fig. 2. The exclusion of Hosaka 2013 in a sensitivity analysis led to a similar estimate of risk reduction, with an IRR of 0.58 (95% CI: 0.49, 0.68). Compared to untreated populations, treated populations had 0.58 times the rate of HCC, shown in Panel A of Fig. 3. An IRD of -0.31 (95% CI: -0.62, 0.00) was found, indicating that among the untreated populations there were 31 excess cases of HCC per 100 person-years of follow up that could be attributed to their treatment status, shown in Panel B of Fig. 3. Despite removing Hosaka 2013, which appeared to contribute to heterogeneity in the Baujat plot, the I² values remained reasonably consistent between the primary and sensitivity analyses (IRR I²: 0% in both analyses; IRD I²: 79% before, 85% after).

## Discussion

Our rigorous meta-analysis highlights the potential benefit of initiating antiviral therapy earlier in CHB disease course. Drawing on four studies suitable for direct comparison and focusing on treatment-naïve individuals as a proxy for earlier-stage CHB, we found an approximate 40% reduction in risk of HCC in treated populations as compared to untreated populations. This meta-analysis addresses a key evidence gap in the absence of long-term RCTs, providing timely insights into the benefits of early antiviral therapy in non-cirrhotic CHB while results from extended follow-up trials such as ATTENTION are awaited. In addition, it also reinforces growing evidence linking prolonged HBV DNA exposure to increased HCC risk and supports existing literature on the benefits of antiviral therapy for individuals with CHB, across the CHB spectrum [75–78]. 

We also demonstrate that the cumulative incidence rates of HCC increased with greater follow-up time, ranging from 0% to 3% at one year and 0% to 30% at 10 years. Of the 50 studies included in the SLR, 44 reported an average follow-up of at least three years and 23 reported at least five years. Our findings suggest a correlation between cumulative exposure to replicating HBV and increased HCC risk. These findings align with evidence from studies published after the initial SLR which would have otherwise met our eligibility criteria [74, 79–90]. 

The clinical importance of preventing progression to HCC is highlighted by the ongoing global burden of primary liver cancer, with national registry data from China demonstrating that liver cancer ranked fourth in incidence and second in cancer mortality in 2022 [91]. Current clinical guidelines recommend antiviral therapy for individuals with non-cirrhotic CHB without risk factors for HCC, primarily based on elevated ALT levels or significant liver fibrosis with high HBV DNA [7]. The 2024 World Health Organization (WHO) HBV guidelines expand indications for CHB treatment but still do not recommend HBV treatment specifically for HCC risk reduction [1]. Several studies have linked HBV viremia to increased HCC risk; however, few have directly compared treated versus untreated individuals to assess the impact of antiviral therapy on HCC risk. In a study by Choi et al. non-cirrhotic patients with high HBV viral load had the lowest HCC risk when treated promptly, while those with moderate viral load had the highest risk, which was not completely mitigated by long-term therapy—suggesting that delayed treatment may lead to irreversible oncogenic changes from prolonged viral replication [76]. Notably, a 4-year interim analysis in the 12-year ATTENTION trial demonstrated that TAF initiation in non-cirrhotic patients with moderate-to-high HBV viral load and normal or mildly elevated ALT concentrations resulted in reduced risk of liver-related serious adverse events, including HCC, compared to those who were untreated [75]. However, the difference was insufficient to stop the trial early. Final results are expected in 2032. Given the established role of DNA damage, mutagenesis, and cancer development [9], these findings collectively underscore the importance of addressing viral DNA integration as part of treatment strategies early in the disease course. This supports evolving treatment guidelines, such as those from EASL, which acknowledge the potential benefit of earlier antiviral therapy in non-cirrhotic populations [2]. Earlier initiation of antiviral therapy may mitigate risk, particularly for younger patients with CHB with low ALT and viral load who may lack consistent clinical follow-up and later develop active disease.

A recent review [77] found a similar HCC risk reduction associated with treatment, with individuals receiving antiviral therapy having a 40–60% lower risk of HCC than untreated individuals, although this study was not limited to NAs or non-cirrhotic individuals and is therefore not directly comparable. Prior to HBV-controlling HIV regimens, HIV co-infection was associated with accelerated progression of HBV-related liver disease compared to mono-infected individuals, including HCC and cirrhosis [92]. The recent study by Lui et al. supports HCC risk reduction associated with antiviral treatment in co-infected individuals, a population often treated earlier in the HBV disease course. However, it is important to note that the participants in this study with HIV co-infection were significantly younger and had a lower percentage of cirrhosis than the HIV-uninfected group [78]. 

This review’s strengths include the use of a predefined protocol, searches of multiple databases and grey literature sources, and a dual-review approach to minimize bias and errors. A particular strength of the evidence base is that hepatic conditions that could be considered confounding factors (such as cirrhosis and other advanced liver conditions) were excluded; this is in contrast with existing meta-analyses which did not exclude studies reporting only on cirrhotic patients or on mixed populations [93, 94]. Additionally, modification of the JBI prevalence checklist to account for the diagnosis of both CHB and HCC conditions ensured a more rigorous evaluation of the diagnosis and reporting quality among studies, enhancing the reliability and validity of our findings. A major strength of the direct comparison meta-analysis was its approach to comparing cohorts within the same studies, allowing for more reliable attribution of HCC risk to treatment effects. Although there were too few studies to adjust for characteristics in a meta-regression, two of the studies [31, 81] employed propensity score matching techniques to balance baseline characteristics between their treated and untreated cohorts. While the inclusion of methodologically heterogeneous studies in the analysis could be considered a limitation in that it may complicate interpretation of the risk reduction associated with treatment, the fact that half of the studies employed propensity score matching is a strength in that it likely led to a greater balance of patient characteristics between the study cohorts than would otherwise be expected in incidence rates derived without propensity score adjustment.

Limitations of the review include the restriction to studies with an abstract or full text in English and the limitation of the evidence base due to the strict eligibility criteria of the review. As most included studies were conducted in Asia, relevant data published in non-English languages may have been missed, potentially influencing effect estimates and limiting regional generalizability. While these criteria were intentional choices given the aim of gathering evidence specific to expanding NA usage in patients with early stages of HBV infection, they limited the number of studies for inclusion. Notably, the requirement of at least three years of NA treatment resulted in the exclusion of some studies focusing only on untreated patients with CHB, limiting the potential of these analyses to compare treated and untreated patients except in the few studies reporting on both treated and untreated cohorts. Consequently, this prevented an adjusted meta-analysis due to the insufficient number of studies. Additionally, there is potential for ascertainment bias in registry data, as treated patients may be more likely to return for follow-up and report symptoms that prompt imaging compared to untreated cohorts.

A limitation of the meta-analysis is that only four studies were suitable for inclusion due to heterogeneity. Most studies did not explicitly report person-time at risk, one of two inputs to the meta-analysis of HCC incidence. For studies that did not report person-time, this value was approximated using the number-at-risk table accompanying the cumulative incidence curve for HCC and multiplying the number of patients at risk by average duration of follow-up, potentially resulting in overestimates of total person-time at risk. Heterogeneity in the direct comparison meta-analysis likely arose from differences in patient inclusion criteria, antivirals tested, or other methodological differences across the studies. Hosaka 2013 [31] and Yuen 2007 [72] both included individuals treated with LAM, an antiviral known to be characterized by high rates of drug resistance [95]. Notably, Hosaka 2013 reported a higher IRR of HCC than the other studies, potentially due to differences in patient and treatment characteristics. The exclusion of Hosaka 2013 in the sensitivity analysis did not alter the estimated risk reduction, indicating a result robust to outliers. However, the narrower confidence intervals suggest increased precision in the estimate potentially due to reduced heterogeneity in the remaining studies. A key limitation of both the SLR and meta-analysis is the relatively limited follow-up time of included studies, as it can take much longer than the several years assessed in many of the included studies for HCC to develop [96]. Additionally, most of the studies are located in Asia, which may restrict the applicability of the findings across diverse populations. Notably, aggressive HCC development has been observed in populations in Sub-Saharan Africa, a group that is largely underrepresented in this review [97]. This underrepresentation emphasized the necessity for further research in diverse regions to better understand the long-term risk and progression of HCC across different populations, particularly since differences in genotype, screening, and access to antiviral therapy that may modify treatment effects have been noted in this population [98–100]. 

In addition, future research should explore differences in HCC risk reduction when antiviral treatment is initiated in early versus late HBV disease stages. Utilizing longitudinal individual patient data to adjust for time since onset of HBV infection and treatment duration would robustly assess the impact of early antiviral treatment on long-term HCC risk; alternatively, it could be used to adjust for repeated measures of viral load, age, and HBeAg status as proxy variables for disease stage. Research should also focus on newer generation antivirals, such as TAF-based regimens. Given the required duration of treatment for inclusion, studies on newer agents were not captured in this SLR. Finally, future research may also investigate the potential benefit of antiviral treatment in reducing the risk of intrahepatic cholangiocarcinoma, which is also associated with CHB [101]. 

This meta-analysis supports the benefits of antiviral therapy in reducing HCC risk among non-cirrhotic individuals with CHB. The analysis did not directly assess HDV DNA integration; however, the observed risk reduction is consistent with broader evidence that viral suppression can lower hepatocarcinogenic risk. Combined with existing literature, these findings further support treatment expansion for non-cirrhotic individuals with CHB to reduce HBV DNA integration early in the disease course and prevent HCC development.

## Supplementary Information

Supplementary Material 1.

## Data Availability

This manuscript utilizes data from publicly available published studies. All data produced in this study are included within the article and its online supplementary materials. For additional inquiries, please contact the corresponding author.

## Abbreviations

AASLD: American Association for the Study of Liver Diseases
AFP: alpha-fetoprotein
ALT: alanine aminotransferase
APASL: Asian Pacific Association for the Study of the Liver
CDSR: Cochrane Database of Systematic Reviews
CENTRAL: Cochrane Central Register of Controlled Trials
CHB: Chronic hepatitis B
CI: confidence interval
CRD: York University Centre for Reviews and Dissemination
EASL: European Association for the Study of the Liver
ETV: entecavir
GPP: Good Publication Practice
HBsAg: hepatitis B surface antigen
HBeAg: hepatitis B e-antigen
HBV: hepatitis B virus
HCC: hepatocellular carcinoma
HCV: hepatitis C virus
HIV: human immunodeficiency virus
HR: Hazard ratio
IARC: International Agency for Research on Cancer
ICD: International Classification of Diseases
IRD: incidence rate difference
IRR: incidence rate ratio
JBI: Joanna Briggs Institute
LAM: lamivudine
LdT: telbivudine
NAs: nucleos(t)ide analogues
NMA: Network meta-analysis
OR: odds ratio
PRISMA: Preferred Reporting Items for Systematic Reviews and Meta-Analyses
RCTs: randomized controlled trials
SLR: systematic literature review
TAF: tenofovir alafenamide
TDF: tenofovir disoproxil fumarate
WHO: World Health Organization
