# Supplementary material for: Hepatocellular carcinoma risk reduction associated with antiviral treatment in patients with noncirrhotic chronic hepatitis B: a systematic literature review and comparative meta-analysis
Source: BMC Gastroenterol. 2026 Jun 26;26:575. doi: 10.1186/s12876-026-04996-y (PMC13584341; doi:10.1186/s12876-026-04996-y)
Supplement: Supplementary file 1 [file 12876_2026_4996_MOESM1_ESM.docx]

**Supplementary Data**

***SLR Methods***

MEDLINE, including MEDLINE In-Process, MEDLINE Epub Ahead of Print, and Embase were searched separately via the Ovid SP platform. The Cochrane Database of Systematic Reviews (CDSR) and the Cochrane Central Register of Controlled Trials (CENTRAL) were searched simultaneously via the Wiley Online platform. The Database of Abstracts and Reviews of Effects (DARE) was searched through the University of York platform.

Following the initial searches, Ovid announced that some Embase records had been missing between January 23, 2023 and February 22, 2023. Since Embase was searched on February 1, 2023 for this SLR, an additional search in Embase using the Ovid platform was conducted on March 13, 2023 to identify any relevant records which were missing from the Ovid platform during the initial searches. These records were subsequently reviewed and included where appropriate (Table S5).

***Meta-Analysis Methods***

Where reported, the number of HCC events was used. For studies that only reported the incidence rate of HCC, and not explicitly the number of HCC events, the number of patients at risk for HCC and the person-time associated with the reported incidence rate were used to approximate the number of HCC events using the following formula:

- number of HCC events = (incidence rate per 100 person-years/100) * number of patients at risk for HCC * mean/median duration of follow up

Alternatively, for studies that did not report the incidence rate or the number of HCC events, but reported Kaplan-Meier curves, the cumulative incidence over the study period was derived using the DigitizeIt software(1) and multiplied by the number of patients at risk at the beginning of the study period to approximate the number of HCC events:

- number of HCC events = cumulative incidence for whole study period * number of patients at risk for HCC

Person-time was used when reported in a study. For studies that did not explicitly report person-time but included Kaplan-Meier curves, the number of patients at risk within each time interval was multiplied by the midpoint of the interval and summed to estimate total person-time.(2)

For studies that did not explicitly report person-time or an incidence rate of HCC, the number of patients at risk for HCC and the mean/median duration of follow-up were used to approximate total person-time, using the following formula:

- total person-time = number of patients at risk for HCC * mean/median duration of follow up

Table S1: Search terms used in Medline (searched via the Ovid SP platform)

| **Term Group** | **#** | **Searches** | **Hits**  **1 Feb 2023** |
| --- | --- | --- | --- |
| **Hepatocellular Carcinoma** | 1 | exp carcinoma, hepatocellular/ | 101905 |
|  | 2 | (((liver cell or hepat$) adj2 carcinoma) or (liver adj cancer$) or (hepat$ adj cancer$) or (liver adj neoplasm$) or hepatoma$ or hepatocarcinoma$ or HCC or "primary liver cancer").ti,ab,kf. | 169475 |
|  | 3 | or/1-2 | 183445 |
| **Interventions** | 4 | exp tenofovir/ or exp telbivudine/ or exp lamivudine/ | 11613 |
|  | 5 | (Tenofovir$ or TAF or TDF or PMPA or vemlidy$ or viread$ or agardon or ictady or tenofera or tenoviral or tenozet or virofob or virten or GS7340 or "GS 7340" or "GS7340 03" or "GS 7340 03" or GS4331 or "GS 4331" or "GS 1275" or GS1275 or CKD390 or "CKD 390" or "GS 1278" or GS1278 or "GS 4331 05" or GS433105 or GSK548470 or "GSK 548470").ti,ab,kf. | 11608 |
|  | 6 | (Entecavir$ or ETV or baraclude$ or entimate or entecabell or enteone or BMS200475 or BMS-200475 or "BMS 200475" or SQ34676 or SQ-34676 or "SQ 34676").ti,ab,kf. | 4428 |
|  | 7 | (Lamivudine$ or epivir$ or hepivir or heptodin or heptovir or inhavir or ladiwin or lamidac or lamivir or slamivudine or victron or zef?ix or 3TC or "BCH 189" or BCH189 or "GR 103665" or GR103665 or GR109714x or "GR 109714x" or "NSC 620753" or NSC620753 or "HSDB 7155" or HSDB7155).ti,ab,kf. | 9858 |
|  | 8 | (Adefovir$ or hepsera or "GS 0393" or GS0393 or GS393 or "GS 393" or PMEA).ti,ab,kf. | 2688 |
|  | 9 | (Telbivudine$ or "LDT 600" or LDT600 or "NV 02B" or NV02B or tyzeka$ or sebivo$ or epavudine).ti,ab,kf. | 775 |
|  | 10 | (Clevudine$ or levovir or fluoromethylarauracil or fluoromethylarauridine or "f mara u" or FMAU).ti,ab,kf. | 291 |
|  | 11 | or/4-10 | 25184 |
| **RCTs** | 12 | Randomized Controlled Trials as Topic/ | 160153 |
|  | 13 | Randomized Controlled Trial/ | 585677 |
|  | 14 | Random Allocation/ | 106902 |
|  | 15 | Double-Blind Method/ | 174155 |
|  | 16 | Single-Blind Method/ | 32451 |
|  | 17 | Placebos/ | 35924 |
|  | 18 | exp Clinical Trials as Topic/ | 380113 |
|  | 19 | Clinical Trial/ | 537000 |
|  | 20 | Clinical Trial, Phase I/ or Clinical Trial, Phase II/ or Clinical Trial, Phase III/ or Clinical Trial, Phase IV/ | 79368 |
|  | 21 | Controlled Clinical Trial/ or Adaptive Clinical Trial/ | 95211 |
|  | 22 | randomized controlled trial.pt. | 585677 |
|  | 23 | clinical trial.pt. | 537000 |
|  | 24 | (clinical trial, phase i or clinical trial, phase ii or clinical trial, phase iii or clinical trial, phase iv).pt. | 79368 |
|  | 25 | (controlled clinical trial or multicenter study).pt. | 421472 |
|  | 26 | (clinical adj trial$).ti,ab,kf. | 476277 |
|  | 27 | ((singl$ or doubl$ or treb$ or tripl$) adj (blind$3 or mask$3)).ti,ab,kf. | 194800 |
|  | 28 | placebo$.ti,ab,kf. | 244300 |
|  | 29 | (allocat$ adj2 random$).ti,ab,kf. | 42735 |
|  | 30 | (Randomi?ed adj2 trial$).ti,ab,kf. | 415680 |
|  | 31 | rct.ti,ab,kf. | 32676 |
|  | 32 | or/12-31 | 1998286 |
| **Observational and RWE studies** | 33 | Epidemiologic Studies/ | 9248 |
|  | 34 | Observational Study/ | 137577 |
|  | 35 | Cohort Studies/ | 324316 |
|  | 36 | exp Case-Control Studies/ | 1387056 |
|  | 37 | Cross-Sectional Studies/ | 454828 |
|  | 38 | Clinical Study/ | 5438 |
|  | 39 | Follow-Up Studies/ | 689559 |
|  | 40 | Longitudinal Studies/ | 162818 |
|  | 41 | Retrospective Studies/ | 1090375 |
|  | 42 | Prospective Studies/ not Randomized Controlled Trials as Topic/ | 642321 |
|  | 43 | (observational adj (study or studies)).ti,ab,kf. | 155518 |
|  | 44 | (cohort adj (study or studies)).ti,ab,kf. | 306258 |
|  | 45 | cohort analy$.ti,ab,kf. | 12116 |
|  | 46 | case control.ti,ab,kf. | 151701 |
|  | 47 | cross sectional.ti,ab,kf. | 492907 |
|  | 48 | (follow up adj (study or studies)).ti,ab,kf. | 57385 |
|  | 49 | longitudinal.ti,ab,kf. | 317472 |
|  | 50 | retrospective.ti,ab,kf. | 717684 |
|  | 51 | (chart adj3 review$).ti,ab,kf. | 52917 |
|  | 52 | exp Registries/ | 115902 |
|  | 53 | (registry or registries).ti,ab,kf. | 171048 |
|  | 54 | (epidemiologic$ adj (study or studies)).ti,ab,kf. | 94650 |
|  | 55 | or/33-54 | 3904939 |
| **Exclusion terms** | 56 | exp animals/ not exp humans/ | 5088371 |
|  | 57 | (comment or editorial or case reports or historical article).pt. | 4080531 |
|  | 58 | (case stud$ or case report$).ti. | 369107 |
|  | 59 | or/56-58 | 9161369 |
| **Combination** | 60 | 3 and 11 | 1351 |
|  | 61 | 32 or 55 | 5342453 |
|  | 62 | 60 and 61 | 583 |
|  | 63 | 62 not 59 | 572 |
|  | 64 | Limit 63 to yr=2002-current | **553** |

**Database:** Ovid MEDLINE(R) and Epub Ahead of Print, In-Process & Other Non-Indexed Citations and Daily 1946 to January 31, 2023.

Table S2: Search terms used in Embase (searched via Ovid SP platform)

| **Term Group** | **#** | **Searches** | **Hits**  **1 Feb 2023** |
| --- | --- | --- | --- |
| **Hepatocellular Carcinoma** | 1 | exp liver cell carcinoma/ or exp liver tumor/ | 336885 |
|  | 2 | (((liver cell or hepat$) adj2 carcinoma) or (liver adj cancer$) or (hepat$ adj cancer$) or (liver adj neoplasm$) or hepatoma$ or hepatocarcinoma$ or HCC or "primary liver cancer").ti,ab,kf. | 237944 |
|  | 3 | or/1-2 | 391354 |
| **Interventions** | 4 | exp tenofovir/ or exp entecavir/ or exp tenofovir alafenamide/ or exp tenofovir disoproxil/ or exp telbivudine/ or exp lamivudine/ or exp adefovir/ or exp clevudine/ | 57039 |
|  | 5 | (Tenofovir$ or TAF or TDF or PMPA or vemlidy$ or viread$ or agardon or ictady or tenofera or tenoviral or tenozet or virofob or virten or GS7340 or "GS 7340" or "GS7340 03" or "GS 7340 03" or GS4331 or "GS 4331" or "GS 1275" or GS1275 or CKD390 or "CKD 390" or "GS 1278" or GS1278 or "GS 4331 05" or GS433105 or GSK548470 or "GSK 548470").ti,ab,kf. | 20829 |
|  | 6 | (Entecavir$ or ETV or baraclude$ or entimate or entecabell or enteone or BMS200475 or BMS-200475 or "BMS 200475" or SQ34676 or SQ-34676 or "SQ 34676").ti,ab,kf. | 8880 |
|  | 7 | (Lamivudine$ or epivir$ or hepivir or heptodin or heptovir or inhavir or ladiwin or lamidac or lamivir or slamivudine or victron or zef?ix or 3TC or "BCH 189" or BCH189 or "GR 103665" or GR103665 or GR109714x or "GR 109714x" or "NSC 620753" or NSC620753 or "HSDB 7155" or HSDB7155).ti,ab,kf. | 15703 |
|  | 8 | (Adefovir$ or hepsera or "GS 0393" or GS0393 or GS393 or "GS 393" or PMEA).ti,ab,kf. | 4668 |
|  | 9 | (Telbivudine$ or "LDT 600" or LDT600 or "NV 02B" or NV02B or tyzeka$ or sebivo$ or epavudine).ti,ab,kf. | 1754 |
|  | 10 | (Clevudine$ or levovir or fluoromethylarauracil or fluoromethylarauridine or "f mara u" or FMAU).ti,ab,kf. | 448 |
|  | 11 | or/4-10 | 68997 |
| **RCTs** | 12 | "randomized controlled trial (topic)"/ | 246833 |
|  | 13 | Randomized Controlled Trial/ | 759524 |
|  | 14 | randomization/ | 97678 |
|  | 15 | double blind procedure/ | 204788 |
|  | 16 | single blind procedure/ | 49801 |
|  | 17 | crossover procedure/ | 73212 |
|  | 18 | placebo/ | 395618 |
|  | 19 | exp "clinical trial (topic)"/ | 421196 |
|  | 20 | Clinical Trial/ | 1064408 |
|  | 21 | phase 1 clinical trial/ or phase 2 clinical trial/ or phase 3 clinical trial/ or phase 4 clinical trial/ | 213231 |
|  | 22 | Controlled Clinical Trial/ or Adaptive Clinical Trial/ or multicenter study/ | 765358 |
|  | 23 | (clinical adj trial$).ti,ab,kf. | 695381 |
|  | 24 | ((singl$ or doubl$ or treb$ or tripl$) adj (blind$3 or mask$3)).ti,ab,kf. | 275656 |
|  | 25 | placebo$.ti,ab,kf. | 359840 |
|  | 26 | (allocat$ adj2 random$).ti,ab,kf. | 53050 |
|  | 27 | (Randomi?ed adj2 trial$).ti,ab,kf. | 564778 |
|  | 28 | rct.ti,ab,kf. | 54270 |
|  | 29 | or/12-28 | 2868069 |
| **Observational and RWE studies** | 30 | Observational Study/ | 304636 |
|  | 31 | cohort analysis/ | 960506 |
|  | 32 | exp case control study/ | 216414 |
|  | 33 | cross-sectional study/ | 527263 |
|  | 34 | Clinical Study/ | 161191 |
|  | 35 | follow up/ | 1965395 |
|  | 36 | longitudinal study/ | 183949 |
|  | 37 | retrospective study/ | 1376455 |
|  | 38 | prospective study/ not "randomized controlled trial (topic)"/ | 824702 |
|  | 39 | (observational adj (study or studies)).ti,ab,kf. | 242227 |
|  | 40 | (cohort adj (study or studies)).ti,ab,kf. | 449319 |
|  | 41 | cohort analy$.ti,ab,kf. | 18644 |
|  | 42 | case control.ti,ab,kf. | 201499 |
|  | 43 | cross sectional.ti,ab,kf. | 642720 |
|  | 44 | (follow up adj (study or studies)).ti,ab,kf. | 75949 |
|  | 45 | longitudinal.ti,ab,kf. | 430886 |
|  | 46 | retrospective.ti,ab,kf. | 1197508 |
|  | 47 | (chart adj3 review$).ti,ab,kf. | 112354 |
|  | 48 | exp disease registry/ | 63641 |
|  | 49 | (registry or registries).ti,ab,kf. | 287031 |
|  | 50 | (epidemiologic$ adj (study or studies)).ti,ab,kf. | 123174 |
|  | 51 | or/30-50 | 5856785 |
| **Exclusion terms** | 52 | ("conference abstract" or "conference review").pt. | 4682589 |
|  | 53 | limit 47 to yr="1974-2020" | 94482 |
|  | 54 | exp animals/ not exp humans/ | 5092950 |
|  | 55 | (comment or editorial or case reports or historical article).pt. | 753285 |
|  | 56 | editorial/ or case report/ or historical article/ | 3560509 |
|  | 57 | (case stud$ or case report$).ti. | 448292 |
|  | 58 | or/52-57 | 12761457 |
| **Combination** | 59 | 3 and 11 | 5329 |
|  | 60 | 29 or 51 | 7839466 |
|  | 61 | 59 and 60 | 3058 |
|  | 62 | 61 not 58 | 1986 |
|  | 63 | Limit 62 to yr=2002-current | **1923** |

**Database:** Embase 1974 to January 31, 2023. On March 13, 2023, an additional search in Embase using the Ovid platform was conducted, only searching records which were missing from the Ovid platform during the initial search date (see Table S5).

Table S3: Search terms used in the Cochrane Library databases (searched simultaneously via the Wiley platform)

| **Term Group** | **#** | **Searches** | **Hits**  **1 Feb 2023** |
| --- | --- | --- | --- |
| **Hepatocellular Carcinoma** | 1 | [mh "carcinoma, hepatocellular"] | 2344 |
|  | 2 | ((("liver cell" or hepat*) NEAR/2 carcinoma) or (liver NEXT cancer*) or (hepat* NEXT cancer*) or (liver NEXT neoplasm*) or hepatoma* or hepatocarcinoma* or HCC or "primary liver cancer"):ti,ab,kw | 8977 |
|  | 3 | {OR #1-#2} | 8977 |
| **Interventions** | 4 | [mh "tenofovir"] | 1424 |
|  | 5 | [mh "telbivudine"] | 122 |
|  | 6 | [mh "lamivudine"] | 1457 |
|  | 7 | (Tenofovir* or TAF or TDF or PMPA or vemlidy* or viread* or agardon or ictady or tenofera or tenoviral or tenozet or virofob or virten or GS7340 or "GS 7340" or "GS7340 03" or "GS 7340 03" or GS4331 or "GS 4331" or "GS 1275" or GS1275 or CKD390 or "CKD 390" or "GS 1278" or GS1278 or "GS 4331 05" or GS433105 or GSK548470 or "GSK 548470"):ti,ab,kw | 4298 |
|  | 8 | (Entecavir* or ETV or baraclude* or entimate or entecabell or enteone or BMS200475 or BMS-200475 or "BMS 200475" or SQ34676 or SQ-34676 or "SQ 34676"):ti,ab,kw | 1071 |
|  | 9 | (Lamivudine* or epivir* or hepivir or heptodin or heptovir or inhavir or ladiwin or lamidac or lamivir or slamivudine or victron or zef?ix or 3TC or "BCH 189" or BCH189 or "GR 103665" or GR103665 or GR109714x or "GR 109714x" or "NSC 620753" or NSC620753 or "HSDB 7155" or HSDB7155):ti,ab,kw | 3489 |
|  | 10 | (Adefovir* or hepsera or "GS 0393" or GS0393 or GS393 or "GS 393" or PMEA):ti,ab,kw | 788 |
|  | 11 | (Telbivudine* or "LDT 600" or LDT600 or "NV 02B" or NV02B or tyzeka* or sebivo* or epavudine):ti,ab,kw | 307 |
|  | 12 | (Clevudine* or levovir or fluoromethylarauracil or fluoromethylarauridine or "f mara u" or FMAU):ti,ab,kw | 61 |
|  | 13 | {OR #4-#12} | 7562 |
| **Combination** | 14 | #3 AND #13 | 262 |
|  | 15 | #14 with Publication Year from 2002 to 2023, in Trials | **254** |
|  | 16 | #14 with Cochrane Library publication date from Jan 2002 to Jan 2023, in Cochrane Reviews | **4** |

**Databases:** CDSR Issue 2 of 12, February 2023; CENTRAL Issue 1 of 12, January 2023.

Table S4: Search terms used in the York CRD platform

| **Term Group** | **#** | **Searches** | **Hits**  **1 Feb 2023** |
| --- | --- | --- | --- |
| **Hepatocellular Carcinoma** | 1 | MeSH DESCRIPTOR carcinoma, hepatocellular EXPLODE ALL TREES | 385 |
|  | 2 | ((("liver cell" or hepat*) adj2 carcinoma) or (liver adj cancer*) or (hepat* adj cancer*) or (liver adj neoplasm*) or hepatoma* or hepatocarcinoma* or HCC or "primary liver cancer") IN ANY FIELD | 858 |
|  | 3 | #1 or #2 | 861 |
| **Interventions** | 4 | MeSH DESCRIPTOR tenofovir EXPLODE ALL TREES or MeSH DESCRIPTOR telbivudine EXPLODE ALL TREES or MeSH DESCRIPTOR lamivudine EXPLODE ALL TREES | 158 |
|  | 5 | (Tenofovir* or TAF or TDF or PMPA or vemlidy* or viread* or agardon or ictady or tenofera or tenoviral or tenozet or virofob or virten or GS7340 or "GS 7340" or "GS7340 03" or "GS 7340 03" or GS4331 or "GS 4331" or "GS 1275" or GS1275 or CKD390 or "CKD 390" or "GS 1278" or GS1278 or "GS 4331 05" or GS433105 or GSK548470 or "GSK 548470") IN ANY FIELD | 95 |
|  | 6 | (Entecavir* or ETV or baraclude* or entimate or entecabell or enteone or BMS200475 or BMS-200475 or "BMS 200475" or SQ34676 or SQ-34676 or "SQ 34676") IN ANY FIELD | 59 |
|  | 7 | (Lamivudine* or epivir$ or hepivir or heptodin or heptovir or inhavir or ladiwin or lamidac or lamivir or slamivudine or victron or zefix or 3TC or "BCH 189" or BCH189 or "GR 103665" or GR103665 or GR109714x or "GR 109714x" or "NSC 620753" or NSC620753 or "HSDB 7155" or HSDB7155) IN ANY FIELD | 198 |
|  | 8 | (Adefovir* or hepsera or "GS 0393" or GS0393 or GS393 or "GS 393" or PMEA) IN ANY FIELD | 57 |
|  | 9 | (Telbivudine* or "LDT 600" or LDT600 or "NV 02B" or NV02B or tyzeka* or sebivo* or epavudine) IN ANY FIELD | 26 |
|  | 10 | (Clevudine* or levovir or fluoromethylarauracil or fluoromethylarauridine or "f mara u" or FMAU) IN ANY FIELD | 1 |
|  | 11 | (#4 or #5 or #6 or #7 or #8 or #9 or #10) | 310 |
| **Combination** | 12 | #3 and #11 | 37 |
|  | 13 | #12 FROM 2002 TO 2015 | 36 |
|  | 14 | #13 IN DARE | **15** |

**Databases:** DARE, Issue 2 of 4, April 2015.

Table S5: Search terms used in Embase, missing records^a^ (searched via the Ovid SP platform)

| **Term Group** | **#** | **Searches** | **Hits**  **13 Mar 2023** |
| --- | --- | --- | --- |
| **Hepatocellular Carcinoma** | 1 | exp liver cell carcinoma/ or exp liver tumor/ | 345190 |
|  | 2 | (((liver cell or hepat$) adj2 carcinoma) or (liver adj cancer$) or (hepat$ adj cancer$) or (liver adj neoplasm$) or hepatoma$ or hepatocarcinoma$ or HCC or "primary liver cancer").ti,ab,kf. | 243461 |
|  | 3 | or/1-2 | 400656 |
| **Interventions** | 4 | exp tenofovir/ or exp entecavir/ or exp tenofovir alafenamide/ or exp tenofovir disoproxil/ or exp telbivudine/ or exp lamivudine/ or exp adefovir/ or exp clevudine/ | 57876 |
|  | 5 | (Tenofovir$ or TAF or TDF or PMPA or vemlidy$ or viread$ or agardon or ictady or tenofera or tenoviral or tenozet or virofob or virten or GS7340 or "GS 7340" or "GS7340 03" or "GS 7340 03" or GS4331 or "GS 4331" or "GS 1275" or GS1275 or CKD390 or "CKD 390" or "GS 1278" or GS1278 or "GS 4331 05" or GS433105 or GSK548470 or "GSK 548470").ti,ab,kf. | 21178 |
|  | 6 | (Entecavir$ or ETV or baraclude$ or entimate or entecabell or enteone or BMS200475 or BMS-200475 or "BMS 200475" or SQ34676 or SQ-34676 or "SQ 34676").ti,ab,kf. | 9037 |
|  | 7 | (Lamivudine$ or epivir$ or hepivir or heptodin or heptovir or inhavir or ladiwin or lamidac or lamivir or slamivudine or victron or zef?ix or 3TC or "BCH 189" or BCH189 or "GR 103665" or GR103665 or GR109714x or "GR 109714x" or "NSC 620753" or NSC620753 or "HSDB 7155" or HSDB7155).ti,ab,kf. | 15825 |
|  | 8 | (Adefovir$ or hepsera or "GS 0393" or GS0393 or GS393 or "GS 393" or PMEA).ti,ab,kf. | 4702 |
|  | 9 | (Telbivudine$ or "LDT 600" or LDT600 or "NV 02B" or NV02B or tyzeka$ or sebivo$ or epavudine).ti,ab,kf. | 1767 |
|  | 10 | (Clevudine$ or levovir or fluoromethylarauracil or fluoromethylarauridine or "f mara u" or FMAU).ti,ab,kf. | 452 |
|  | 11 | or/4-10 | 70074 |
| **RCTs** | 12 | "randomized controlled trial (topic)"/ | 255219 |
|  | 13 | Randomized Controlled Trial/ | 774227 |
|  | 14 | randomization/ | 98382 |
|  | 15 | double blind procedure/ | 208212 |
|  | 16 | single blind procedure/ | 51047 |
|  | 17 | crossover procedure/ | 74453 |
|  | 18 | placebo/ | 400111 |
|  | 19 | exp "clinical trial (topic)"/ | 433930 |
|  | 20 | Clinical Trial/ | 1070283 |
|  | 21 | phase 1 clinical trial/ or phase 2 clinical trial/ or phase 3 clinical trial/ or phase 4 clinical trial/ | 217228 |
|  | 22 | Controlled Clinical Trial/ or Adaptive Clinical Trial/ or multicenter study/ | 775800 |
|  | 23 | (clinical adj trial$).ti,ab,kf. | 712143 |
|  | 24 | ((singl$ or doubl$ or treb$ or tripl$) adj (blind$3 or mask$3)).ti,ab,kf. | 279620 |
|  | 25 | placebo$.ti,ab,kf. | 365189 |
|  | 26 | (allocat$ adj2 random$).ti,ab,kf. | 54142 |
|  | 27 | (Randomi?ed adj2 trial$).ti,ab,kf. | 579761 |
|  | 28 | rct.ti,ab,kf. | 55705 |
|  | 29 | or/12-28 | 2920643 |
| **Observational and RWE studies** | 30 | Observational Study/ | 319418 |
|  | 31 | cohort analysis/ | 1006877 |
|  | 32 | exp case control study/ | 221951 |
|  | 33 | cross-sectional study/ | 549583 |
|  | 34 | Clinical Study/ | 162410 |
|  | 35 | follow up/ | 2015427 |
|  | 36 | longitudinal study/ | 189869 |
|  | 37 | retrospective study/ | 1433815 |
|  | 38 | prospective study/ not "randomized controlled trial (topic)"/ | 849448 |
|  | 39 | (observational adj (study or studies)).ti,ab,kf. | 250772 |
|  | 40 | (cohort adj (study or studies)).ti,ab,kf. | 466811 |
|  | 41 | cohort analy$.ti,ab,kf. | 19305 |
|  | 42 | case control.ti,ab,kf. | 206144 |
|  | 43 | cross sectional.ti,ab,kf. | 663272 |
|  | 44 | (follow up adj (study or studies)).ti,ab,kf. | 77065 |
|  | 45 | longitudinal.ti,ab,kf. | 441357 |
|  | 46 | retrospective.ti,ab,kf. | 1233205 |
|  | 47 | (chart adj3 review$).ti,ab,kf. | 114664 |
|  | 48 | exp disease registry/ | 65644 |
|  | 49 | (registry or registries).ti,ab,kf. | 294837 |
|  | 50 | (epidemiologic$ adj (study or studies)).ti,ab,kf. | 124705 |
|  | 51 | or/30-50 | 6020019 |
| **Exclusion terms** | 52 | ("conference abstract" or "conference review").pt. | 4711128 |
|  | 53 | limit 47 to yr="1974-2020" | 94565 |
|  | 54 | exp animals/ not exp humans/ | 5145731 |
|  | 55 | (comment or editorial or case reports or historical article).pt. | 767785 |
|  | 56 | editorial/ or case report/ or historical article/ | 3608312 |
|  | 57 | (case stud$ or case report$).ti. | 457267 |
|  | 58 | or/52-57 | 12887815 |
| **Combination** | 59 | 3 and 11 | 5471 |
|  | 60 | 29 or 51 | 8031126 |
|  | 61 | 59 and 60 | 3152 |
|  | 62 | 61 not 58 | 2072 |
|  | 63 | limit 62 to yr=2002-current | 2009 |
|  | 64 | limit 63 to restoredrecords | **54** |

**Database:** Embase 1974 to March 10, 2023. **Footnotes:** ^a^ In the weeks following Embase searches, Ovid had announced that Embase records had been missing from any searches conducted between January 23^,^ 2023, when records initially went missing, and February 22, 2023, when records were officially restored. Therefore, it was recommended that once these records were restored, searches be re-run to ensure any missing records were then captured. On March 13, 2023, an additional search in Embase using the Ovid platform was conducted, only searching records which were missing from the Ovid platform during the initial search date, as reported above. These records were subsequently reviewed and included where appropriate.

Table S6: Search terms used for congresses

| **Congress** | **Searches** | **Search Date** | **Hits** |
| --- | --- | --- | --- |
| **AASLD 2021** | tenofovir, entecavir, adefovir, telbivudine, clevudine, lamivudine, analog, and analogue | 23, February 2023 | 144 |
| **AASLD 2022** | tenofovir, entecavir, adefovir, telbivudine, clevudine, lamivudine, analog, and analogue | 23, February 2023 | 94 |
| **EASL International Liver Congress 2021** | tenofovir, entecavir, adefovir, telbivudine, clevudine, lamivudine, analog, and analogue | 20, February 2023 | 79 |
| **EASL International Liver Congress 2022** | tenofovir, entecavir, adefovir, telbivudine, clevudine, lamivudine, analog, and analogue | 20, February 2023 | 140 |
| **EASL Viral Hepatitis Elimination Meeting 2022** | tenofovir, entecavir, adefovir, telbivudine, clevudine, lamivudine, analog, and analogue | 20, February 2023 | 4 |
| **APASL 2022** | tenofovir, entecavir, adefovir, telbivudine, clevudine, lamivudine, analog, and analogue | 20, February 2023 | 9 |
| **CROI 2021** | tenofovir, entecavir, adefovir, telbivudine, clevudine, lamivudine, analog, and analogue | 20, February 2023 | 70 |
| **CROI 2022** | tenofovir, entecavir, adefovir, telbivudine, clevudine, lamivudine, analog, and analogue | 20, February 2023 | 78 |

**Abbreviations:** AASLD, American Association for the Study of Liver Diseases; APASL, Asia Pacific Association for the Study of the Liver; CROI, Conference on Retroviruses and Opportunistic Infections; EASL, European Association for the Study of the Liver.

Table S7: Eligibility criteria for the SLR

| **Domain** | **Inclusion Criteria** | **Exclusion Criteria** |
| --- | --- | --- |
| **Patient Population** | Children or adult patients with HBV, in populations treated or untreated with NAs (not mixed cohorts)  Subpopulations of interest, including but not limited to:   - Comorbidities and/or coinfections, such as HIV and HCV - Viral status, such as HBeAg, HBsAg, and HBV DNA detection - Time to treatment initiation (early vs late in disease course) | - Patients with other conditions - Patients with HDV co-infection - Patients with liver cirrhosis reported at baseline |
| **Intervention/ Comparator** | Studies reporting NA treatment with mean or median treatment duration of ≥3 years, with any of the following interventions:   - Tenofovir alafenamide (TAF)/Vemlidy® - Tenofovir disoproxil fumarate (TDF)/Viread® - Entecavir (ETV)/Baraclude® - Telbivudine (LdT)/Tyzeka® - Adefovir Dipivoxil/Hepsera® - Clevudine/Levovir® - Lamivudine/Epivir-HBV/Zeffix/Heptodin® | - All other interventions, including interferons - Studies reporting a mean or median NA treatment duration <3 years |
| **Comparator** | Any eligible intervention or no treatment | Not applicable |
| **Outcomes** | Incidence of HCC | Any other outcome |
| **Study Design** | - Randomized controlled studies - Non-randomized interventional studies - Observational studies, including: - Prospective or retrospective cohort - Cross sectional - Epidemiological - SLRs and (N)MAs considered relevant at the title/abstract review stage will be included and hand-searched for primary studies. They will be excluded at the full text review stage | All other study designs |
| **Publication Type** | - Peer-reviewed studies - Conference abstracts (published 2021 or later) | - Commentaries - Editorials - Conference abstracts (published 2020 or earlier) |
| **Language** | Articles in English | Articles not in English |
| **Other** | Articles published in 2002 or later | Articles published in 2001 or earlier |

**Abbreviations:** DNA, deoxyribonucleic acid; HBeAg, hepatitis B e-antigen; HBsAg, hepatitis B surface antigen; HBV, hepatitis B virus; HCC, hepatocellular carcinoma; HCV, hepatitis C virus; HIV, human immunodeficiency virus; NA, nucleo(s/t)ide analog; (N)MAs, (network) meta-analyses; SLR, systematic literature review.

Fig. S1: PRISMA diagram from the SLR

**Abbreviations:** CDSR, Cochrane Database of Systematic Reviews; CENTRAL, Cochrane Central Register of Controlled Trials; DARE, Database of Abstracts of Reviews of Effects; SLR, systematic literature review.

Table S8: Summary of study quality as assessed by the JBI prevalence checklist

| Study | Q1. Was the sample frame appropriate to address the target population? | Q2. Were study participants sampled in an appropriate way? | Q3. Was the sample size adequate? | Q4. Were the study subjects and the setting described in detail? | Q5. Was the data analysis conducted with sufficient coverage of the identified sample? | *Q6a. Were valid methods used for the identification of HBV infection? | *Q6b. Were valid methods used for the identification of HCC development? | *Q7a. Was HBV infection measured in a standard, reliable way for all participants? | *Q7b. Was HCC development measured in a standard, reliable way for all participants? | Q8. Was there appropriate statistical analysis? | Q9. Was the response rate adequate, and if not, was the low response rate managed appropriately? |
| --- | --- | --- | --- | --- | --- | --- | --- | --- | --- | --- | --- |
| **Ando 2018** | **N** | **N** | **Y** | **N** | **Y** | **U** | **Y** | **U** | **Y** | **NA** | **NA** |
| **Chiu 2020** | **N** | **N** | **Y** | **Y** | **Y** | **U** | **U** | **U** | **U** | **NA** | **NA** |
| **Cho 2014** | **N** | **N** | **Y** | **N** | **Y** | **Y** | **Y** | **Y** | **Y** | **Y** | **NA** |
| **Choi 2020** | **Y** | **N** | **Y** | **N** | **Y** | **U** | **Y** | **Y** | **U** | **Y** | **NA** |
| **Choi 2022a** | **Y** | **U** | **Y** | **Y** | **Y** | **U** | **Y** | **U** | **Y** | **Y** | **NA** |
| **Choi 2022b** | **Y** | **U** | **Y** | **N** | **U** | **U** | **U** | **U** | **U** | **Y** | **U** |
| **Chun 2021** | **N** | **U** | **Y** | **Y** | **Y** | **Y** | **Y** | **U** | **U** | **N** | **NA** |
| **Dezanet 2021** | **Y** | **U** | **Y** | **Y** | **N** | **U** | **U** | **Y** | **U** | **NA** | **N** |
| **Hosaka 2010** | **N** | **N** | **Y** | **N** | **Y** | **U** | **Y** | **U** | **Y** | **Y** | **NA** |
| **Hosaka 2013** | **Y** | **N** | **Y** | **N** | **Y** | **Y** | **Y** | **Y** | **N** | **Y** | **NA** |
| **Hosaka 2022** | **N** | **N** | **Y** | **N** | **Y** | **Y** | **Y** | **Y** | **Y** | **N** | **NA** |
| **Ji 2022** | **Y** | **N** | **Y** | **N** | **Y** | **Y** | **Y** | **Y** | **Y** | **U** | **Y** |
| **Kim 2015** | **N** | **N** | **Y** | **N** | **U** | **Y** | **Y** | **U** | **N** | **Y** | **U** |
| **Kim 2017a** | **Y** | **N** | **Y** | **N** | **Y** | **N** | **Y** | **Y** | **Y** | **Y** | **NA** |
| **Kim 2017b** | **N** | **N** | **Y** | **N** | **Y** | **Y** | **Y** | **Y** | **N** | **Y** | **NA** |
| **Kim 2018** | **Y** | **N** | **Y** | **Y** | **Y** | **Y** | **Y** | **U** | **Y** | **Y** | **NA** |
| **Ko 2019** | **Y** | **N** | **Y** | **N** | **U** | **Y** | **Y** | **Y** | **Y** | **NA** | **Y** |
| **Kobashi 2011** | **Y** | **U** | **Y** | **N** | **U** | **Y** | **Y** | **Y** | **Y** | **NA** | **U** |
| **Kozuka 2019** | **Y** | **N** | **Y** | **Y** | **Y** | **U** | **Y** | **U** | **Y** | **Y** | **NA** |
| **Lampertico 2007** | **Y** | **N** | **Y** | **N** | **U** | **U** | **U** | **U** | **U** | **NA** | **U** |
| **Lee 2016** | **Y** | **N** | **Y** | **N** | **Y** | **Y** | **Y** | **Y** | **Y** | **Y** | **NA** |
| **Lee 2019** | **N** | **U** | **Y** | **N** | **Y** | **U** | **U** | **Y** | **U** | **NA** | **Y** |
| **Lee 2021** | **Y** | **U** | **Y** | **N** | **Y** | **Y** | **Y** | **U** | **U** | **Y** | **NA** |
| **Lee 2022** | **Y** | **Y** | **Y** | **N** | **Y** | **U** | **Y** | **U** | **Y** | **Y** | **NA** |
| **Li 2017** | **Y** | **N** | **Y** | **N** | **U** | **Y** | **Y** | **Y** | **U** | **NA** | **U** |
| **Lim 2014** | **Y** | **N** | **Y** | **Y** | **Y** | **N** | **Y** | **N** | **Y** | **N** | **NA** |
| **Lin 2019** | **Y** | **N** | **Y** | **N** | **Y** | **U** | **Y** | **U** | **Y** | **Y** | **NA** |
| **Moon 2015** | **Y** | **N** | **Y** | **N** | **Y** | **Y** | **Y** | **Y** | **Y** | **Y** | **NA** |
| **Nagata 2016** | **N** | **N** | **Y** | **Y** | **Y** | **U** | **Y** | **U** | **Y** | **N** | **NA** |
| **Niro 2013** | **Y** | **N** | **Y** | **Y** | **U** | **U** | **Y** | **U** | **U** | **NA** | **U** |
| **Oh 2020** | **Y** | **Y** | **Y** | **N** | **Y** | **Y** | **Y** | **Y** | **Y** | **Y** | **NA** |
| **Orito 2015** | **Y** | **U** | **Y** | **N** | **Y** | **U** | **Y** | **U** | **Y** | **NA** | **NA** |
| **PAGE-B Cohort** | **Y** | **Y** | **Y** | **N** | **N** | **Y** | **Y** | **Y** | **Y** | **N** | **Y** |
| **Papatheodoridis 2015** | **Y** | **Y** | **Y** | **N** | **Y** | **Y** | **Y** | **N** | **N** | **Y** | **NA** |
| **Papatheodoridis 2017** | **Y** | **Y** | **Y** | **Y** | **Y** | **Y** | **Y** | **Y** | **Y** | **Y** | **Y** |
| **Papatheodoridi 2022** | **Y** | **N** | **N** | **Y** | **Y** | **Y** | **Y** | **Y** | **U** | **Y** | **NA** |
| **Pellicelli 2014** | **Y** | **Y** | **Y** | **Y** | **Y** | **Y** | **Y** | **N** | **U** | **N** | **NA** |
| **RETRACT-B** | **N** | **U** | **Y** | **N** | **Y** | **U** | **U** | **N** | **U** | **NA** | **Y** |
| **Shim 2017** | **N** | **N** | **Y** | **Y** | **Y** | **U** | **Y** | **U** | **Y** | **N** | **NA** |
| **Shin 2018** | **Y** | **N** | **Y** | **N** | **Y** | **Y** | **Y** | **U** | **Y** | **Y** | **NA** |
| **Sou 2020** | **Y** | **U** | **Y** | **N** | **Y** | **U** | **Y** | **U** | **Y** | **NA** | **NA** |
| **Tseng 2017** | **Y** | **Y** | **Y** | **N** | **U** | **U** | **Y** | **U** | **Y** | **N** | **U** |
| **Wang 2016** | **Y** | **N** | **Y** | **N** | **Y** | **U** | **Y** | **U** | **Y** | **NA** | **NA** |
| **Wong 2013** | **N** | **N** | **Y** | **Y** | **U** | **U** | **Y** | **Y** | **N** | **Y** | **U** |
| **Wu 2017** | **Y** | **N** | **Y** | **N** | **Y** | **U** | **Y** | **U** | **Y** | **Y** | **NA** |
| **Yamada 2015** | **Y** | **Y** | **Y** | **Y** | **Y** | **U** | **Y** | **U** | **Y** | **Y** | **NA** |
| **Yang 2013** | **Y** | **N** | **Y** | **N** | **Y** | **U** | **Y** | **U** | **Y** | **Y** | **NA** |
| **Yang 2014** | **N** | **N** | **Y** | **Y** | **Y** | **U** | **Y** | **Y** | **U** | **NA** | **NA** |
| **Yip 2020** | **Y** | **Y** | **Y** | **N** | **Y** | **Y** | **Y** | **Y** | **Y** | **Y** | **NA** |
| **Yuen 2007** | **Y** | **N** | **Y** | **Y** | **U** | **U** | **Y** | **U** | **N** | **NA** | **U** |

**Footnotes:** *JBI prevalence checklist has been modified to account for the diagnosis of HBV infection and HCC in the patient population.

**Abbreviations:** N, no; NA, not applicable; U, unclear; Y, yes.

Table S9: Summary of HCC surveillance and diagnostic criteria

| **Study** | **Surveillance Frequency** | **Surveillance Details** | **Diagnostic Guidelines** | **Diagnosis** |
| --- | --- | --- | --- | --- |
| **Ando 2018(3)** | 1−3 months | AFP and DCP tumor markers | AASLD | Dynamic CT, dynamic MRI, or contrast-enhanced ultrasonography according to findings of hypervascularity in arterial phase and washout in the early or delayed venous phase in dynamic CT or dynamic MRI |
| **Chiu 2020(4)** | NR | NR | NR | NR |
| **Cho 2014(5)** | 3–6 months | Liver biopsy or imaging modalities including CT, MRI and/or digital subtraction angiography; tumor markers (e.g., AFP, PIVKA-II) | Korean Liver Cancer Study Group | NR |
| **Choi 2020(6)** | 6 months | Liver ultrasonography; serum AFP | NR* | Histologically or by using non-invasive diagnostic criteria |
| **Choi 2022a(7)** | 6 months | Liver ultrasonography; serum AFP | AASLD; EASL | Histologic examination or typical imaging findings (nodule >1 cm with arterial hypervascularity and portal/delayed-phase washout) on either dynamic CT or MRI |
| **Choi 2022b(8)** | NR | NR | NR | NR |
| **Chun 2021(9)** | NR | NR | NR | NR (HCC identified by ICD-10 code C22) |
| **Dezanet 2021(10)** | NR | NR | NR | NR |
| **Hosaka 2010**  **(All patients) (11)** | Monthly | Tumor makers including AFP and PIVKA-II | NR | Hypervascularity on angiography and/or histological examination, characteristic features of CT, MRI and ultrasonography |
| **Hosaka 2010 (Continued LAM treatment cohort) (11)** | 6 months | NR | NR | Abdominal ultrasonography |
| **Hosaka 2013(12)** | 6–12 months | Ultrasonography or helical dynamic CT | NR | Predominantly imaging, including dynamic CT, MRI, and/or digital subtraction angiography. When the hepatic nodule did not show typical imaging features, diagnosis was confirmed by fine-needle aspiration biopsy followed by histological examination |
| **Hosaka 2022(13)** | 6–12 months | Ultrasonography; helical dynamic CT; MRI | NR | Predominantly imaging, including dynamic CT, MRI, and/or digital subtraction angiography |
| **Ji 2022(14)** | ≤12 weeks | Ultrasonography; AFP | NR | Multi-phase dynamic contrast-enhanced MRI |
| **Kim 2015(15)** | NR | NR | NR* | NR |
| **Kim 2017a(16)** | 6 months | Ultrasonography; serum AFP | AASLD; EASL | Histologic examination and/or typical image patterns (nodule >1 cm with arterial hypervascularity and portal/delayed-phase washout) by dynamic CT and/or MRI |
| **Kim 2017b(17)** | 3–6 months or more frequently as required for at least 1 year | NR | Korean Liver Cancer Study Group | Histological evaluation or by clinical imaging |
| **Kim 2018(18)** | 3–6 months | Ultrasonography; laboratory workups, including AFP and PIVKA-II | AASLD | Dynamic CT and/or MRI |
| **Ko 2019(19)** | 6 months | Ultrasonography; AFP | NR* | Contrast imaging with CT or MRI or by histology from surgical specimens |
| **Kobashi 2011(20)** | 6 months | AFP and DCP were measured alternately every month; image diagnosis by ultrasonography, dynamic enhanced CT or dynamic enhanced MRI was performed every 6 months | Liver Cancer Study Group of Japan | If elevation of AFP or DCP was observed, imaging diagnosis was performed within a month. HCC was confirmed by the finding of enhanced arterial contrast uptake followed by washout in the portal venous phase and equivalent phase by dynamic enhanced CT or dynamic enhanced MRI, or histologically by fine-needle tumor biopsy |
| **Kozuka 2019(21)** | 3–6 months | Ultrasonography or dynamic CT and/or MRI | NR | Presence of arterial hypervascularization and delayed washout on dynamic CT and/or MRI |
| **Lampertico 2007(22)** | NR | NR | NR | NR |
| **Lee 2016(23)** | 6 months | Abdominal ultrasonography and/or contrast-enhanced multiphase CT; serum AFP | AASLD | MRI or liver biopsy |
| **Lee 2019(24)** | NR | NR | NR | NR |
| **Lee 2021(25)** | NR | NR | NR | NR (HCC identified by ICD-10 codes) |
| **Lee 2022(26)** | 6 months | Ultrasonography; serum AFP | EASL | NR |
| **Li 2017(27)** | NR | NR | NR | Elevated AFP and characteristic imaging acquired using CT, MRI or both |
| **Lim 2014(28)** | 6 months | Ultrasonography; serum AFP | AASLD | Dynamic CT and/or MRI. When the hepatic nodule did not show imaging features typical of HCC, a biopsy specimen was taken and subjected to pathologic examination |
| **Lin 2019(29)** | 1−3 months | NR | NR | Ultrasonography and helical dynamic CT; presence of typical hypervascular characteristics on angiography, in addition to the findings of dynamic CT. If no typical findings of HCC were observed, the diagnosis was confirmed based on the Li-RADS and finally confirmed by fine needle aspiration biopsy followed by histological examination |
| **Moon 2015(30)** | NR | NR | EASL | Typical radiologic features of HCC or histological confirmation |
| **Nagata 2016(31)** | 6 months | NR | NR | Ultrasonography |
| **Niro 2013(32)** | NR | NR | NR | Contrast imaging with CT scan and/or magnetic resonance second-generation contrast ultrasound examinations or at histology in those who consented to liver biopsy |
| **Oh 2020(33)** | 3–6 months | Abdominal sonography or CT; serum AFP | NR | NR |
| **Orito 2015(34)** | At least 6 months | Ultrasound, CT, or MRI | NR | NR |
| **PAGE-B Cohort(35)** | ≤12 months (non-cirrhotic patients); ≤6 months (cirrhotic patients) | Ultrasonography with or without AFP | EASL | Standard histological and/or compatible radiological findings |
| **Papatheodoridis 2015(36)** | Yearly (all non-cirrhotic patients in 5 centers; only for patients with a family history of HCC in 2 centers) | Ultrasonography | NR | Standard histological and/or compatible radiological findings |
| **Papatheodoridis 2017(37)** | ≤12 months (non-cirrhotic patients); ≤6 months (cirrhotic patients) | Ultrasonography with or without AFP | EASL | Standard histological and/or compatible radiological findings |
| **Papatheodoridi 2022(38)** | 6 months | Ultrasonography with or without AFP | NR | Histological findings and/or compatible radiological findings by CT or MRI |
| **Pellicelli 2014(39)** | NR | NR | EASL | Histological findings or by one or two concordant imaging results (CT or NMR) compatible with HCC depending on the dimensions of the nodule |
| **RETRACT-B(40)** | NR | NR | NR | NR |
| **Shim 2017(41)** | 6 months | Ultrasonography; serum AFP | AASLD | Presence of typical enhancement patterns in dynamic radiologic tests or histologic examination |
| **Shin 2018(42)** | 3–6 months | Ultrasonography and laboratory workups, including AFP and PIVKA-II | AASLD | Dynamic CT and/or MRI |
| **Sou 2020(43)** | 3–6 months | Abdominal ultrasonography; serum AFP | AASLD | NR |
| **Tseng 2017(44)** | 3–6 months (liver function and AFP); 6 months (ultrasonography) | Abdominal ultrasonography; serum liver function tests; AFP | AASLD | Histology/cytology or by typical imaging findings (arterial enhancement and venous wash-out by contrast-enhanced CT or MRI scanning) in hepatic nodules larger than 1 cm |
| **Wang 2016(45)** | 3–6 months | Liver ultrasonography | AASLD | NR |
| **Wong 2013(46)** | 1–2 years or more frequently if AFP level increased to ≥20 µg/L | Abdominal ultrasonography | AASLD | Histopathologic confirmation, detection of a positive lesion with at least 2 imaging techniques (trans-abdominal ultrasonography, triphasic CT, MRI, or hepatic angiography), or detection with one imaging technique coupled with an AFP concentration ≥400 ng/mL |
| **Wu 2017(47)** | 3–6 months | Liver ultrasonography | AASLD | NR |
| **Yamada 2015(48)** | 3–6 months | Ultrasonography of the abdomen; CT; MRI | NR | Presence of typical hypervascular characteristics evident on the CT/MRI scans. If no typical signs of HCC were observed, either hepatic angiography or fine-needle aspiration biopsy was performed with the patient’s consent, or the patient was carefully followed until a diagnosis was possible on the basis of a definite observation |
| **Yang 2013(49)** | 3–6 months | Liver ultrasonography | NR | Histopathological findings of tumor tissues; or one typical HCC feature on a dynamic image or serum AFP levels >200 ng/mL if the nodule was >2 cm in cirrhotic liver; or two typical HCC features on dynamic images if the nodule was between 1 and 2 cm in a cirrhotic liver |
| **Yang 2014(50)** | NR | NR | NR | Contrast-enhanced CT or MRI |
| **Yip 2020(51)** | 6–12 months | Ultrasound; AFP | NR | HCC was identified based on ICD-9-CM diagnosis codes (155.0 HCC and 155.2) or procedure codes for HCC treatment |
| **Yuen 2007(52)** | 6 months | AFP measurement, followed by ultrasound and CT if AFP was increased | NR | Histology or typical features on imaging |

**Footnotes:** *Studies reported following unspecified guidelines.
**Abbreviations:** AASLD, American Association for the Study of Liver Diseases; AFP, alpha-fetoprotein; CT, computed tomography; DCP, des-gamma-carboxy prothrombin; EASL, European Association for the Study of the Liver; HBV, hepatitis B virus; HCC, hepatocellular carcinoma; ICD(-9/10)(-CM), International Classification of Diseases (- Ninth Revision/Tenth Revision) (- Clinical Modification); MRI, magnetic resonance imaging; NR, not reported; PIVKA-II, protein induced by vitamin K absence-II.

Supplementary References

1.	DigitizeIt. DigitizeIt Germany [Available from: <https://www.digitizeit.xyz/>.

2.	Clark LJ, Zolotor A, Curteis T. MSR154 Comparison of Methods to Estimate Total Person-Time at Risk for Synthesis Studies of Incidence Rates in Observational Data. Value in Health. 2024;27(12):S468.

3.	Ando Y, Ishigami M, Ishizu Y, Kuzuya T, Honda T, Hayashi K, et al. Cumulative incidence and risk factors for the development of hepatocellular carcinoma in patients with chronic hepatitis B who achieved sustained disappearance of viremia by nucleos(t)ide analog treatment. Hepatology Research. 2018;48(3):E240-E51.

4.	Chiu SM, Kuo YH, Wang JH, Hung CH, Hu TH, Lu SN, et al. Associations of HBV Genotype B vs C Infection With Relapse After Cessation of Entecavir or Tenofovir therapy. Clinical gastroenterology and hepatology : the official clinical practice journal of the American Gastroenterological Association. 2020;27.

5.	Cho JY, Paik YH, Sohn W, Cho HC, Gwak GY, Choi MS, et al. Patients with chronic hepatitis B treated with oral antiviral therapy retain a higher risk for HCC compared with patients with inactive stage disease. Gut. 2014;63(12):1943-50.

6.	Choi J, Kim GA, Han S, Lim YS. Earlier Alanine Aminotransferase Normalization During Antiviral Treatment Is Independently Associated With Lower Risk of Hepatocellular Carcinoma in Chronic Hepatitis B. American Journal of Gastroenterology. 2020;115(3):406-14.

7.	Choi WM, Kim GA, Choi J, Han S, Lim YS. Increasing on-treatment hepatocellular carcinoma risk with decreasing baseline viral load in HBeAg-positive chronic hepatitis B. Journal of Clinical Investigation. 2022;132(10) (no pagination).

8.	Choi WM, Yip TCF, Wong GLH, Yee LJ, Curteis T, Clark LJ, et al. Baseline Viral Load Is Associated with the on-Treatment Risk of Hepatocellular Carcinoma in Non-Cirrhotic, Hbeag-Positive Chronic Hepatitis B Patients: Results from an Individual Patient Data Meta-Analysis from Three Countries. Hepatology. 2022;76:S305-S6.

9.	Chun HS, Park S, Lee M, Cho Y, Kim HS, Choe AR, et al. Association of physical activity with the risk of hepatocellular carcinoma in patients with chronic hepatitis b. Cancers. 2021;13(14) (no pagination).

10.	Dezanet LNC, Miailhes P, Lascoux-Combe C, Chas J, Maylin S, Gabassi A, et al. Profiles of liver fibrosis evolution during long-term tenofovir treatment in HIV-positive patients coinfected with hepatitis B. Liver International. 2021;41(12):2874-84.

11.	Hosaka T, Suzuki F, Kobayashi M, Hirakawa M, Kawamura Y, Yastuji H, et al. Development of HCC in patients receiving adefovir dipivoxil for lamivudine-resistant hepatitis B virus mutants. Hepatol Res. 2010;40(2):145-52.

12.	Hosaka T, Suzuki F, Kobayashi M, Seko Y, Kawamura Y, Sezaki H, et al. Long-term entecavir treatment reduces hepatocellular carcinoma incidence in patients with hepatitis B virus infection. Hepatology. 2013;58(1):98-107.

13.	Hosaka T, Suzuki F, Fujiyama S, Kawamura Y, Sezaki H, Akuta N, et al. Ultrasensitive Assay for Hepatitis B Core-Related Antigen Predicts Hepatocellular Carcinoma Incidences During Entecavir. Hepatology Communications. 2022;6(1):36-49.

14.	Ji D, Chen Y, Dong Z, Xiao XH, Yang Y. Incidence of hepatocellular carcinoma reduced in chronic hepatitis B patients treated with entercavir plus Biejia-Ruangan: extended follow-up of a multicenter, randomised, double-blinded, placebo-controlled trial. Journal of hepatology. 2022;77:S377‐S8.

15.	Kim WR, Loomba R, Berg T, Aguilar Schall RE, Yee LJ, Dinh PV, et al. Impact of long-term tenofovir disoproxil fumarate on incidence of hepatocellular carcinoma in patients with chronic hepatitis B. Cancer. 2015;121(20):3631-8.

16.	Kim GA, Han S, Kim HD, An J, Lim YS. Higher risk of hepatocellular carcinoma in chronic hepatitis B vs chronic hepatitis C after achievement of virologic response. Journal of Viral Hepatitis. 2017;24(11):990-7.

17.	Kim JH, Sinn DH, Kang W, Gwak GY, Paik YH, Choi MS, et al. Low-level viremia and the increased risk of hepatocellular carcinoma in patients receiving entecavir treatment. Hepatology. 2017;66(2):335-43.

18.	Kim BG, Park NH, Lee SB, Lee H, Lee BU, Park JH, et al. Mortality, liver transplantation and hepatic complications in patients with treatment-naive chronic hepatitis B treated with entecavir vs tenofovir. Journal of Viral Hepatitis. 2018;25(12):1565-75.

19.	Ko KL, To WP, Mak LY, Seto WK, Ning Q, Fung J, et al. A large real-world cohort study examining the effects of long-term entecavir on hepatocellular carcinoma and HBsAg seroclearance. Journal of viral hepatitis. 2019;21.

20.	Kobashi H, Miyake Y, Ikeda F, Yasunaka T, Nishino K, Moriya A, et al. Long-term outcome and hepatocellular carcinoma development in chronic hepatitis B or cirrhosis patients after nucleoside analog treatment with entecavir or lamivudine. Hepatology Research. 2011;41(5):405-16.

21.	Kozuka R, Enomoto M, Sato-Matsubara M, Yoshida K, Motoyama H, Hagihara A, et al. Association between HLA-DQA1/DRB1 polymorphism and development of hepatocellular carcinoma during entecavir treatment. Journal of Gastroenterology and Hepatology (Australia). 2019;34(5):937-46.

22.	Lampertico P, Vigano M, Manenti E, Iavarone M, Sablon E, Colombo M. Low Resistance to Adefovir Combined With Lamivudine: A 3-Year Study of 145 Lamivudine-Resistant Hepatitis B Patients. Gastroenterology. 2007;133(5):1445-51.

23.	Lee J, Yoo SH, Sohn W, Kim HW, Choi YS, Won JH, et al. Obesity and hepatocellular carcinoma in patients receiving entecavir for chronic hepatitis B. Clin Mol Hepatol. 2016;22(3):339-49.

24.	Lee HW, Park JY, Kim SG, Tak WY, Yim HJ, Jang BK, et al. Long-term Outcomes of Antiviral Therapy in Patients With Advanced Chronic HBV Infection. Clinical Gastroenterology and Hepatology. 2019;17(13):2811-3.e1.

25.	Lee J, Kim GA, Kim HJ, Cho S, Ko MJ, Lim YS. Tenofovir Disoproxil Fumarate on the Risk of Hepatocellular Carcinoma in Chronic Hepatitis B Patients with Failure to Preceding Treatments: A Nationwide Cohort Study. Journal of viral hepatitis. 2021;01.

26.	Lee JH, Shin SK, Kang SH, Kim TH, Yim HJ, Yim SY, et al. Long-Term Prediction Model for Hepatocellular Carcinoma in Patients with Chronic Hepatitis B Receiving Antiviral Therapy: Based on Data from Korean Patients. Journal of Clinical Medicine. 2022;11(22) (no pagination).

27.	Li ZQ, Hu CL, Yu P, Gu XY, Zhang JJ, Li H, et al. The development of hepatocarcinoma after long-term antivirus treatment of Chinese patients with chronic hepatitis B virus infection: Incidence, long-term outcomes and predictive factors. Clinics and Research in Hepatology and Gastroenterology. 2017;41(3):311-8.

28.	Lim YS, Han S, Heo NY, Shim JH, Lee HC, Suh DJ. Mortality, liver transplantation, and hepatocellular carcinoma among patients with chronic hepatitis B treated with entecavir vs lamivudine. Gastroenterology. 2014;147(1):152-61.

29.	Lin CC, Bair MJ, Liu CY, Lin ZY, Chen CJ, Chen MJ, et al. High percentage atypical hepatocellular carcinoma in chronic hepatitis B patients treated with nucleos(t)ide analogs. Medicine (United States). 2019;98(1):E13818.

30.	Moon JC, Kim SH, Kim IH, Lee CH, Kim SW, Lee SO, et al. Disease progression in chronic hepatitis B patients under long-term antiviral therapy. Gut and Liver. 2015;9(3):395-404.

31.	Nagata N, Kagawa T, Hirose S, Arase Y, Tsuruya K, Anzai K, et al. Off-treatment durability of antiviral response to nucleoside analogues in patients with chronic hepatitis B. BMC Gastroenterol. 2016;16:38.

32.	Niro GA, Ippolito AM, Fontana R, Valvano MR, Gioffreda D, Iacobellis A, et al. Long-term outcome of hepatitis B virus-related Chronic Hepatitis under protracted nucleos(t)ide analogues. Journal of Viral Hepatitis. 2013;20(7):502-9.

33.	Oh H, Yoon EL, Jun DW, Ahn SB, Lee HY, Jeong JY, et al. No Difference in Incidence of Hepatocellular Carcinoma in Patients With Chronic HBV Infection Treated With Entecavir vs Tenofovir. Clinical gastroenterology and hepatology : the official clinical practice journal of the American Gastroenterological Association. 2020;02.

34.	Orito E, Hasebe C, Kurosaki M, Osaki Y, Joko K, Watanabe H, et al. Risk of hepatocellular carcinoma in cirrhotic hepatitis B virus patients during nucleoside/nucleotide analog therapy. Hepatology Research. 2015;45(8):872-9.

35.	Papatheodoridis GV, Sypsa V, Dalekos GN, Yurdaydin C, Van Boemmel F, Buti M, et al. Hepatocellular carcinoma prediction beyond year 5 of oral therapy in a large cohort of Caucasian patients with chronic hepatitis B. Journal of hepatology. 2020;22.

36.	Papatheodoridis GV, Dalekos GN, Yurdaydin C, Buti M, Goulis J, Arends P, et al. Incidence and predictors of hepatocellular carcinoma in Caucasian chronic hepatitis B patients receiving entecavir or tenofovir. Journal of Hepatology. 2015;62(2):363-70.

37.	Papatheodoridis GV, Idilman R, Dalekos GN, Buti M, Chi H, van Boemmel F, et al. The risk of hepatocellular carcinoma decreases after the first 5 years of entecavir or tenofovir in Caucasians with chronic hepatitis B. Hepatology. 2017;66(5):1444-53.

38.	Papatheodoridi M, Su TH, Hadziyannis E, Liao CH, Orfanidou A, Yang HC, et al. Hepatocellular carcinoma after treatment cessation in non-cirrhotic HBeAg-negative chronic hepatitis B: A multicentre cohort study. Liver International. 2022;42(3):541-50.

39.	Pellicelli AM, Vignally P, Messina V, Izzi A, Mazzoni E, Barlattani A, et al. Long term nucleotide and nucleoside analogs treatment in chronic hepatitis B HBeAg negative genotype D patients and risk for hepatocellular carcinoma. Ann Hepatol. 2014;13(4):376-85.

40.	Hirode G, Choi HSJ, Chen CH, Su TH, Seto WK, Van Hees S, et al. Off-Therapy Response After Nucleos(t)ide Analogue Withdrawal in Patients With Chronic Hepatitis B: An International, Multicenter, Multiethnic Cohort (RETRACT-B Study). Gastroenterology. 2022;162(3):757-71.e4.

41.	Shim JJ, Oh CH, Kim JW, Lee CK, Kim BH. Liver cirrhosis stages and the incidence of hepatocellular carcinoma in chronic hepatitis B patients receiving antiviral therapy. Scandinavian Journal of Gastroenterology. 2017;52(9):1029-36.

42.	Shin JW, Jung SW, Lee SB, Lee BU, Park BR, Park EJ, et al. Medication Nonadherence Increases Hepatocellular Carcinoma, Cirrhotic Complications, and Mortality in Chronic Hepatitis B Patients Treated With Entecavir. American Journal of Gastroenterology. 2018;113(7):998-1008.

43.	Sou FM, Hu TH, Hung CH, Lai HC, Wang JH, Lu SN, et al. Incidence and predictors of hepatocellular carcinoma beyond year 5 of entecavir therapy in chronic hepatitis B patients. Hepatology International. 2020;14(4):513-20.

44.	Tseng TC, Liu CJ, Su TH, Yang WT, Chen CL, Yang HC, et al. Fibrosis-4 Index Helps Identify HBV Carriers with the Lowest Risk of Hepatocellular Carcinoma. American Journal of Gastroenterology. 2017;112(10):1564-74.

45.	Wang HM, Hung CH, Lee CM, Lu SN, Wang JH, Yen YH, et al. Three-year efficacy and safety of tenofovir in nucleos(t)ide analog-naive and nucleos(t)ide analog-experienced chronic hepatitis B patients. J Gastroenterol Hepatol. 2016;31(7):1307-14.

46.	Wong GL, Chan HL, Chan HY, Tse PC, Tse YK, Mak CW, et al. Accuracy of risk scores for patients with chronic hepatitis B receiving entecavir treatment. Gastroenterology. 2013;144(5):933-44.

47.	Wu IT, Hu TH, Hung CH, Lu SN, Wang JH, Lee CM, et al. Comparison of the efficacy and safety of entecavir and tenofovir in nucleos(t)ide analogue-naive chronic hepatitis B patients with high viraemia: a retrospective cohort study. Clinical Microbiology and Infection. 2017;23(7):464-9.

48.	Yamada R, Hiramatsu N, Oze T, Morishita N, Harada N, Yakushijin T, et al. Impact of alpha-fetoprotein on hepatocellular carcinoma development during entecavir treatment of chronic hepatitis B virus infection. J Gastroenterol. 2015;50(7):785-94.

49.	Yang SC, Lee CM, Hu TH, Wang JH, Lu SN, Hung CH, et al. Virological response to entecavir reduces the risk of liver disease progression in nucleos(t)ide analogue-experienced HBV-infected patients with prior resistant mutants. Journal of Antimicrobial Chemotherapy. 2013;68(9):2154-63.

50.	Yang YW, Lee CY, Hu RH, Lee PH, Tsai MK. Long-term effects of prophylactic and therapeutic lamivudine treatments in hepatitis B surface antigen-positive renal allograft recipients. Clinical and Experimental Nephrology. 2014;18(1):144-50.

51.	Yip TC, Wong VW, Chan HL, Tse YK, Lui GC, Wong GL. Tenofovir Is Associated With Lower Risk of Hepatocellular Carcinoma Than Entecavir in Patients With Chronic HBV Infection in China. Gastroenterology. 2020;158(1):215-25 e6.

52.	Yuen MF, Seto WK, Chow DH, Tsui K, Wong DK, Ngai VW, et al. Long-term lamivudine therapy reduces the risk of long-term complications of chronic hepatitis B infection even in patients without advanced disease. Antivir Ther. 2007;12(8):1295-303.
